# Beyond Sol-Gel: Molecular Gels with Different Transitions

**DOI:** 10.3390/gels9040273

**Published:** 2023-03-25

**Authors:** Senem Yilmazer, Duncan Schwaller, Philippe J. Mésini

**Affiliations:** Institute Charles Sadron, University of Strasbourg, CNRS, 23 Rue du Loess, 67000 Strasbourg, France

**Keywords:** molecular gels, organogels, transitions, polymorphism, phase diagrams, crystallization, syneresis

## Abstract

The existence of sol–gel transitions is one of the most manifest properties of molecular gels. These transitions reflect their nature since they correspond to the association or dissociation of low weight molecules through non-covalent interactions to form the network constitutive of the gel. Most described molecular gels undergo only one gel-to-sol transition upon heating, and the reverse sol-to-gel transition upon cooling. It has been long observed that different conditions of formation could lead to gels with different morphologies, and that gels can undergo a transition from gel to crystals. However, more recent publications report molecular gels which exhibit additional transitions, for instance gel-to-gel transitions. This review surveys the molecular gels for which, in addition to sol–gel transitions, transitions of different nature have been reported: gel-to-gel transitions, gel-to-crystal transition, liquid–liquid phase separations, eutectic transformations, and synereses.

## 1. Introduction

Molecular gels or organogels are physical gels resulting from the self-association in different solvents of low molecular weight molecules called organogelators or LMWG (low molecular weight gelators) [1,2,3,4,5,6,7,8,9,10,11,12,13,14]. Since the beginning of the 1990s, they have received increasing interest and now constitute an active field of research in soft matters. The association of the gelator molecules proceeds through non-covalent bonds (hydrogen bonds, π-π stacking, Van der Waals, etc.) and is generally thermoreversible. The resulting aggregates, usually with high aspect ratio, form a 3D network and endow the mixtures with their viscoelastic properties. Many gelators have been discovered and constitute a large library of compounds with a wide diversity of chemical structures from simple alkanes [15] to elaborate peptides [16]. Much of this research has been triggered by potential applications in different fields: drug delivery and tissue engineering [16,17,18,19,20,21], food applications [22,23,24,25,26], cosmetics [27,28], oil spill recovery [29,30], or electronic devices [31,32].

The existence of reversible sol–gel transitions is the most prominent property of organogels since it defines them. It is often by observing such transition during a heating/cooling cycle that new gelators are discovered. Organogels are most often characterized by quantities related to this transition: the minimal gel concentration, which is the required concentration to observe the sol-to-gel transition, or the values of the gel-to-sol transition temperatures *T*_GS_, which indicates the range of temperature of the thermodynamical stability of the gels. The variations of *T*_GS_ with the concentration of gelator *c* define the boundary between two domains in the phase diagram: the sol and the gel. Most of the published diagrams of organogelators show these two domains only; this simplicity stands in contrast with the diagrams in other domains, such as metallurgy or polymer science [32,33,34,35]. The phase behaviors of the gels are in reality more complex. Their apparent simplicity may be explained by the low range of the explored concentrations in gelator. For instance, at a low temperature, the crystallization of the solvent, when the eutectic is reached, should be systematically observed. However, since the resulting solid has no practical interest, low temperatures are seldom explored. The studies are also often restricted to low concentrations because of the efficiency of the gelator: low amounts are sufficient to reach the viscoelastic properties sought for applications. However, at higher concentrations, the phase behavior may be more complex. Other transitions may appear, and consequently, other domains in the phase diagrams. Some articles and reviews [3,36,37] have already discussed the possibility of more complex phase diagrams for organogelators.

In recent years, more authors have reported that, in addition to the classical gel-to-sol transitions, other kinds of transitions, for instance gel-to-gel transitions, even at the low concentrations, where organogels are usually studied. These transitions directly impact the properties of the gels and their applications. The goal of the present paper is to review organogels for which there is evidenced transitions distinct from the sol-gel ones. This paper will survey only transitions induced thermally or by ageing. The transitions involving a chemical, photochemical transformation, or complexation of the gelator are beyond the scope of this review. These transitions, induced by different stimuli [38,39,40], light [41], pH, redox reactions [42], anions [43,44,45,46], analytes [47], or enzymes [48] have been already thoroughly reviewed; moreover, many of them are gel–sol transitions. The present review will examine gel-to-gel transitions, gel-to-crystal transitions, liquid–liquid phase separations, eutectic transitions, and syneresis. Whenever it has been studied, the impact of such transitions on the phase diagram will be also reported.

## 2. Gel–Gel Transitions

### 2.1. Thermoreversible Transitions

**Figure 1:**
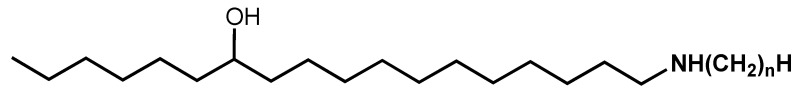
Chemical formula of the **HSN-*n*** (*n* = 0–5) family.

The series of compounds **HSN-*n*** (Figure 1) form thermoreversible gels in CCl_4_ [49]. Gels of **HSN-2** and **HSN-3** in CCl_4_ with concentrations up to 5 wt% are turbid at 24 °C. Above a given temperature, these gels transform into transparent gels. Rheological experiments show that below this temperature, the samples have a solid-like behavior, with *G*′ higher than *G*″. Above this temperature, *G*′ decreases but remains higher than *G*″, which confirms that the sample is still a gel. When heated at higher temperatures, the transparent gels eventually transform into a sol.

**Figure 2:**
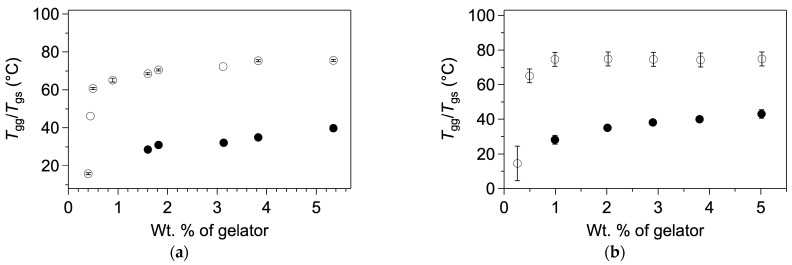
Phase diagram of (**a**) **HSN-2**/CCl_4_ and (**b**) **HSN-3**/CCl_4_. ○ Gel-to-sol transition (*T*_GS_); • gel-to-gel transition (*T*_GG_). Reprinted with permission from Ref. [49]. Copyright 2011 American Chemical Society.

The gel-to-gel transition is also observable by DSC, both during the heating and cooling phases, which proves it is thermoreversible. DSC also showed the same gel-to-gel transition for **HSN-4** gels in CCl_4_, although no visual change occurred. The phase diagrams of **HSN-2** (Figure 2a) and **HSN-3** (Figure 2b) in CCl_4_ were established by DSC and inverted tube tests. In both systems, the first transition is present for concentrations between 1 wt% and 5 wt%.

For **HSN-2** and **HSN-3**, the gel-to-gel transition corresponds to a strong change of the texture visible by optical microscopy (OM). For **HSN-3**, the opaque gels contain large fibrils and spherulitic patterns, whereas the clear gels show no visible structure by OM. For the gels of **HSN-4**, POM shows only a slight change in texture during the transition (although the transition is proved by DSC). The gels above and below the transition have different X-ray diffraction patterns, which indicates a different molecular packing. FTIR studies also show a different H-bonding pattern. Therefore, both gels correspond to different polymorphs. According to the model proposed by the authors, in the gel at low temperature, CCl_4_ molecules are intercalated in the fibers or in the fiber bundles. Upon heating, the hydrogen bonds pattern changes, which leads to a different molecular packing and the expulsion of the CCl_4_ molecules.

**Figure 3:**
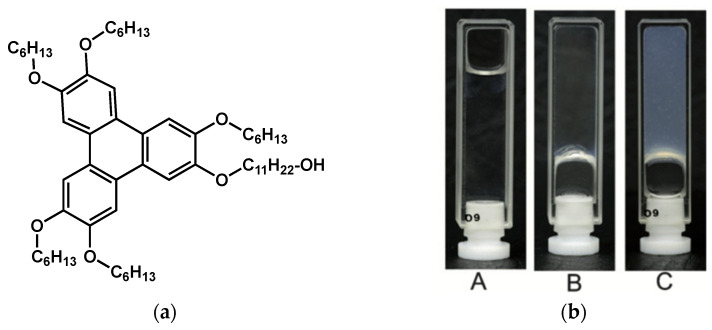
(**a**) Molecular structure of the amphiphilic triphenylene **A**. (**b**) Aspect of the organogels of **A** in 90/10 ethanol/water (4.3 mmol/L). (**A**): at 50 °C, liquid sample; (**B**): at 36 °C, transparent soft gel; (**C**): at 27 °C, turbid hard gel. Reproduced from ref. [50] with permission from the Royal Society of Chemistry.

The asymmetrical hexaether of triphenylene **A** (Figure 3a) gels pure ethanol and ethanol/water mixtures [50]. This organogelator was studied in 90/10 ethanol/water.

The compound is solubilized at 60 °C (Figure 3(bA)), and when the solution is cooled, two transitions take place. The first one, at 41 °C, is the sol-to-gel transition, and yields a transparent gel (Figure 3(bB)). The second one, at 35 °C, is the transition of this gel into a turbid gel (Figure 3(bC)).

Dynamical mechanical analysis of the sample above, between and below the transitions, reveals three different mechanical behaviors. At high temperature, the sample is liquid. Between 35 °C and 41 °C, the transparent gel has a shear modulus of ∼10 Pa and is named soft gel. Below 35 °C, the turbid gel shows a shear modulus of ∼10^4^ Pa and is named hard gel. Therefore, the gel-to-gel transition is clearly identified by a change in mechanical properties from a soft gel to a hard gel.

The morphology of the gel was studied by AFM. Both the soft and hard gels are composed of fibers with no significant difference in sizes and shapes. When analyzed by WAXS, the hard gel shows broad peaks corresponding to distances of 25 Å and 14 Å. The soft gel shows only the broad scattering intensities, close to those of the liquids. These observations show that the fibers in the hard gel have a higher crystalline order than in the soft gel, which may explain a higher elastic modulus.

In conclusion, for both **A**/EtOH-H_2_O and **HSN-*n***/CHCl_3_ systems, the gel-to-gel transition corresponds to a different crystalline order.

**Figure 4:**
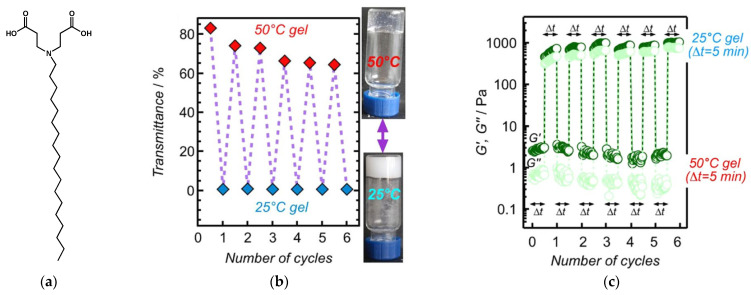
(**a**): Structure of the amphiphile **C18ADPA**. (**b**): transmittance of **C18ADPA**/water gels (20 mg/mL) during heating/cooling cycles; (**c**): elastic and viscous moduli of the same gel during the cycles. Reproduced form ref. [51].

Xie et al. have observed a gel-to-gel transition in a hydrogel of **C18ADPA** (Figure 4a) [51]. The gel forms at pH < 5.5. At 50 °C, the gel is translucent with a transmittance comprised between 60% and 90%, and at 25 °C, it becomes opaque with a null transmittance. (Figure 4b). The elastic and viscous moduli *G*′ and *G*″ were measured for the samples at both temperatures (Figure 4c). For both samples, *G*′ > *G*″, which confirmed their solid-like behaviors. However, in the translucent gel, *G*’ has a value of ∼10 Pa, two orders of magnitude lower than the for turbid gel (*G*′ ∼1000 Pa). The variations of the transmittances and the moduli could be reproduced during several heating and cooling cycles, which clearly shows the gel-to-gel transition is reversible.

**Figure 5:**
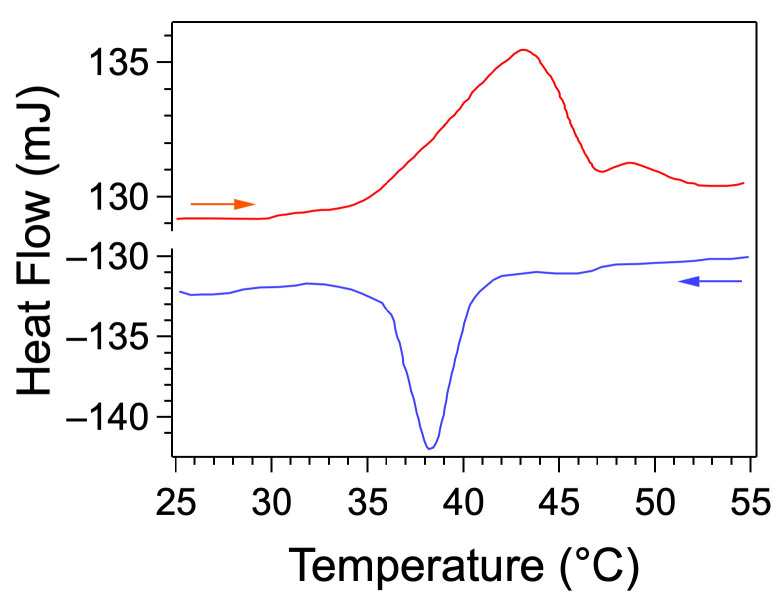
DSC of **C18ADPA**/water gels (20 mg/mL) during heating/cooling cycles (2 °C/min) (data from reference [51]).

DSC thermograms were measured between 25 °C and 55 °C (Figure 5). A major endotherm, with a maximum observed at 43 °C, corresponds to the gel-to-gel transition. A minor peak is also observed at 49 °C, assigned by the authors to an order-disorder transition involving the alkyl chains. Throughout this range of temperatures, *G*′ remains greater than *G*″, which confirms that the sample remains in the gel state. Upon cooling, the exotherm at 38 °C corresponds to the reverse transition.

**Figure 6:**
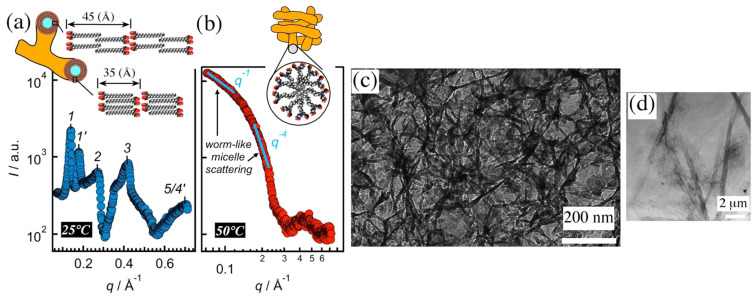
XRD, cryo-TEM, and TEM measurement of the **C18ADPA** hydrogel (2 wt%, pH = 5.37). XRD and proposed model of the structure of the gel (**a**) at 25 °C, crystalline bilayers, (**b**) at 50 °C, worm-like micelles, (**c**) cryo-TEM image of the gel at 50 °C, (**d**) TEM image of the gel at 25 °C. Adapted from ref. [51].

The structure and the morphology of both gels were determined by a combination of XRD, cryo-TEM, and TEM (Figure 6). At 25 °C, the gel is composed of microtubules (Figure 6d). SEM studies showed that these tubes grow branches from their cross-sections. According to the authors, the walls of the tubes are made up of lamellar structures composed of bilayers, either weakly interdigitated alkyl-chains (distance = 45 Å) or fully interdigitated chains (d = 35 Å) (Figure 6a).

At 50 °C, cryo-TEM micrographs (Figure 6c) showed the presence of worm-like micelles, consistent with the X-ray intensities at low angles (Figure 6b), varying with the power law *I*~*q*^−1^.

**Figure 7:**
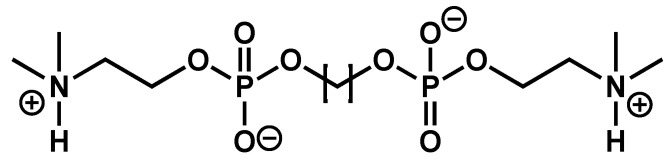
Chemical structure of **Me_2_PE-C*n*-Me_2_PE**.

Meister et al. have studied the series of bolaamphiphiles **Me_2_PE-C*n*-Me_2_PE** [52,53,54] (Figure 7), which gel aqueous solutions at pH 5. At this pH, the polar heads are under their zwitterionic form.

**Figure 8:**
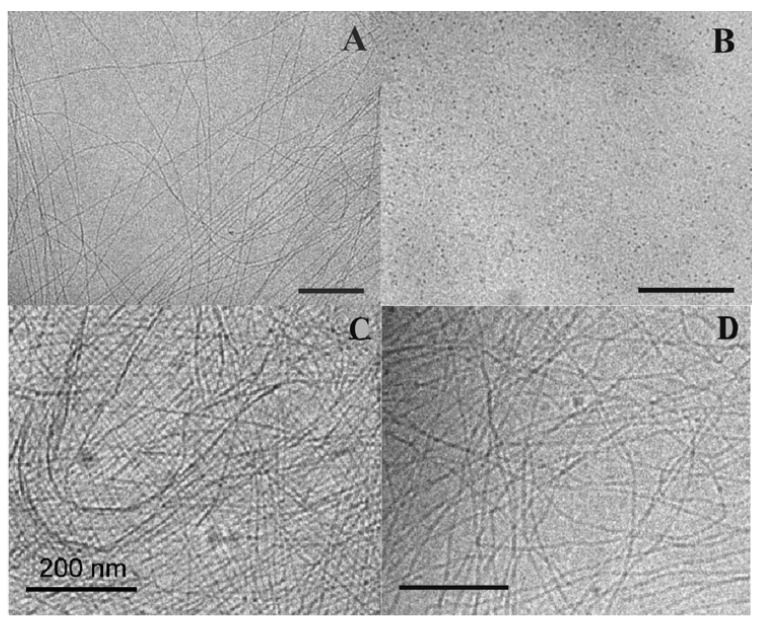
Cryo-electron micrographs of 1 mg/mL suspensions of **Me_2_PE-C*n*-Me_2_PE** in solution at pH 5, quenched from temperature below or above *T*_m_1. (**A**): *n* = 24, 20 °C showing fibers; (**B**): *n* = 24, 35 °C (between *T*_m_1 and *T*_m_2, see Figure 9), micelles; (**C**): *n* = 28, 20 °C, fibers I; (**D**): *n* = 28, 45 °C (between *T*_m_1 and *T*_m_1′, see Figure 9), fibers II. Bar = 200 nm. Reprinted with permission from Ref. [54]. Copyright 2008 American Chemical Society.

At 20 °C, these compounds self-assemble into fibrils with widths of a few nm and lengths of several micrometers. For the derivatives with 22 carbons, 24 carbons, and 26 carbons, the thermograms measured by DSC exhibit 2 endotherms upon heating, with the maximum at the temperatures of *T*_m_1 and *T*_m_2. CryoTEM and SANS experiments show that below *T*_m_1 the derivatives self-assemble into fibrillar structures (Figure 8A). Above *T*_m_1, the fibrils transform into micellar aggregates (Figure 8B). The second transition, at *T*_m_2 does not change the morphology of the aggregates: the transition corresponds to the transformation of micelles, named micelles I, to another kind of micelles, named micelles II.

For derivatives with more than 27 carbons, 3 endotherms are observed in DSC experiments, at temperatures *T*_m_1, *T*_m_1′, and *T*_m_2, respectively. Below *T*_m_1 and between *T*_m_1 and *T*_m_1′, the presence of fibers is observed by cryo-TEM and by SANS experiments (Figure 8C,D). For the C_32_ derivative, below *T*_m_1, rheological measurements show that the system is a gel; with *G*′ > *G*″. At *T*_m_1, its viscoelastic properties change, with both *G*′ and *G*″ decreasing, but it remains a gel [53]. The fibers below and above *T*_m_1, named fibers I and fibers II, respectively, have the same morphology, but are obviously different since they are interconverted by a first-order transition. Their inner structure was studied by temperature variable FTIR. The transition between fibers I and II corresponds to a change in the stretching and scissoring CH_2_ bands [52]. Therefore, the transition involves an increase in the alkyl chain disorder with more gauche conformations. At *T*_m_2, the same bands show weak shifts, which show that the transformation from micelles I to micelles II also involves an increase in the conformational disorder of the alkyl chains.

**Figure 9:**
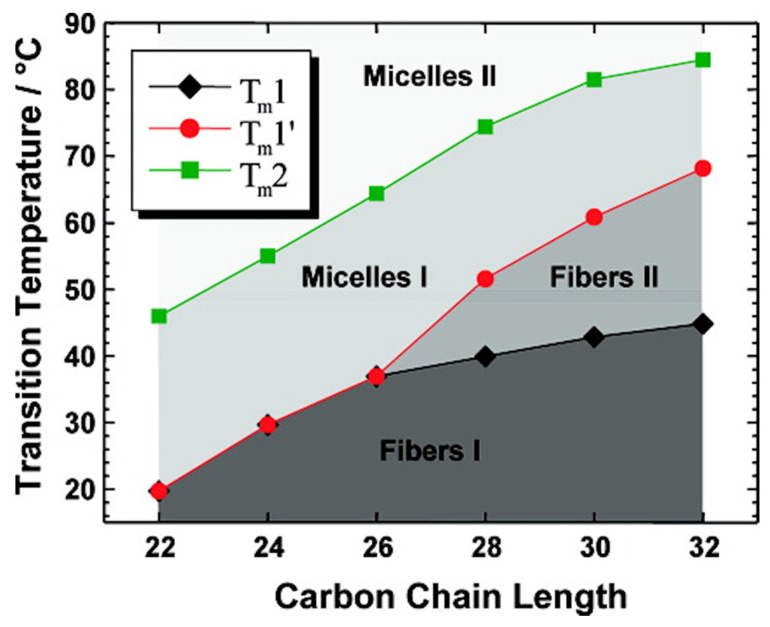
Stability domains of **Me_2_PE-C*n*-Me_2_PE** at pH 5 (*c* = 1 mg/mL) as a function of the chain length *n*. The red line represents the gel-to-sol transition; the black line between fibers I and II represents a gel-to-gel transition. Reprinted with permission from Ref. [54]. Copyright 2008 American Chemical Society.

The different temperatures measured by DSC enable the authors to map a phase diagram (Figure 9) showing the domains of stability of each species as a function of the alkyl chain length. In this diagram, the gel-to-sol transition is represented by the red line and corresponds to the formation of micelles I either from fibers I (for *n* ≤ 26) or fibers II (for *n* ≥ 28). The black line, the boundary between fibers I and II (for *n* ≥ 28), represents a gel-to-gel transition. Finally, this diagram shows an example of sol-to-sol transition, represented by the green line, with the transformation from micelles I to micelles II.

**Figure 10:**
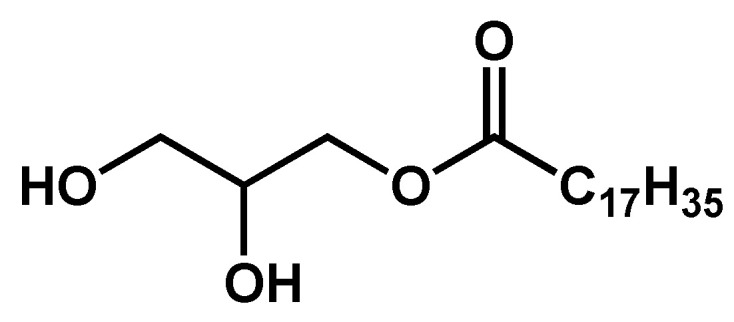
Chemical formula of the C18 monoglyceride **MG**.

The C18 monoglyceride (**MG**) (Figure 10) forms gels in hazelnut oil. The phase behavior of these mixtures was studied by Chen et al. [55].

**Figure 11:**
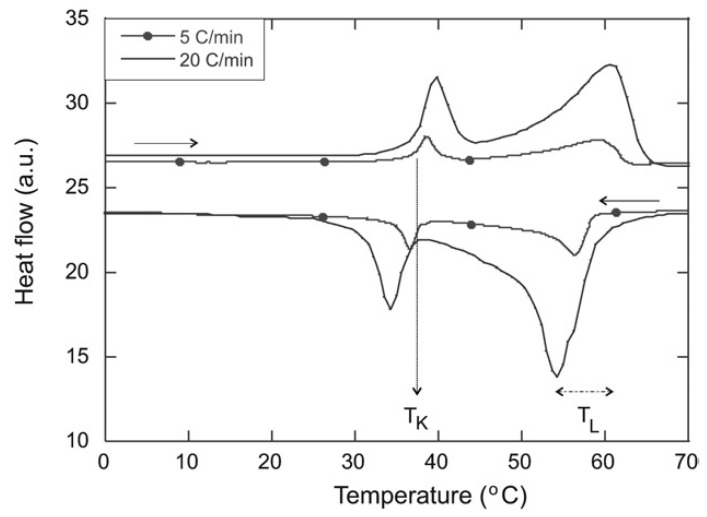
Heating and cooling scan 10 wt% **MG**/oil mixture at 5 and 20 °C/min. Both heating and cooling phases show two transitions at temperature *T*_K_ and *T*_L_. Reproduced from ref. [55] with permission from the Royal Society of Chemistry.

For concentrations above 2 wt%, the DSC clearly shows two endothermic transitions (Figure 11) upon heating and the symmetrical exothermic transitions upon cooling.

The rheological behavior of the mixture was studied as a function of temperature. Upon cooling close to *T*_L_, it shows a sol-to-gel transition: the elastic and the viscous moduli increasing abruptly, and the elastic modulus becomes higher than the viscous modulus. Close to *T*_K_, the lower transition temperature, the elastic modulus slightly decreases, but remains greater than the viscous modulus: the sample transits, but remains a gel with its solid-like behavior.

**Figure 12:**
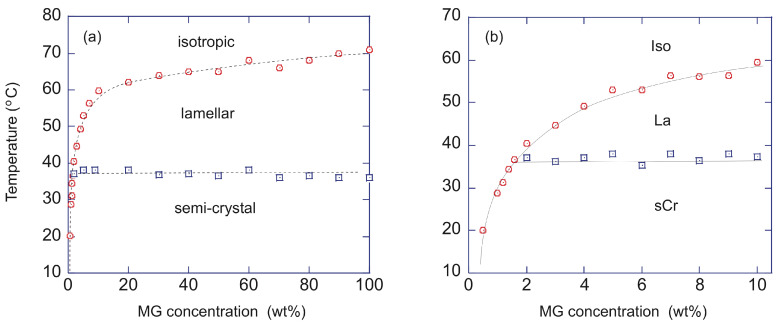
Phase diagram of **MG**/hazelnut oil gel. Boundaries of the three phases upon cooling: isotropic fluid, inverse lamellar, and the sub-alpha crystalline phases. (**a**): full diagram, (**b**) enlargement. Circles: gel-to-sol transitions; squares: gel-to-gel transitions. Reproduced from ref. [55] with permission from the Royal Society of Chemistry.

DSC experiments allowed to map the phase diagram for the full range of concentrations (Figure 12a). Above the concentration of 2 wt%, two transitions are observed (Figure 12b). The transition at higher temperature corresponds to the transition of the sol to a gel made of a lamellar phase (La). The second transition, at a lower temperature, corresponds to the transition from the lamellar gel to another gel, made of a solid phase named sub-alpha crystalline or semi-crystalline phase (sCr). The transition at low temperatures is non-variant because, during this transition, three phases (isotropic, lamellar, and semi-crystal) are in equilibrium. There are two constituents and temperature is the only physical intensive parameter that can be changed. Therefore, according to Gibbs’ phase rule, the variance is zero.

**Figure 13:**
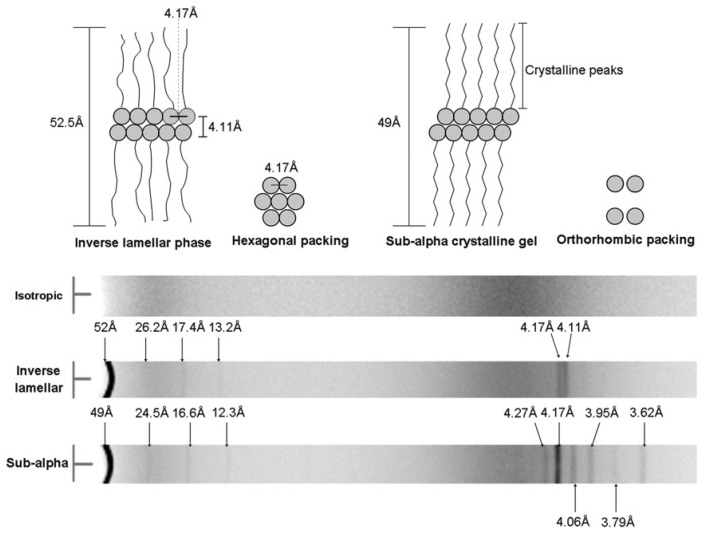
Diagram of the X-ray scattering peaks of the different phases. Reproduced from ref. [55] with permission from the Royal Society of Chemistry.

In oil, **MG** forms an inverse lamellar phase. Indeed, at 45 °C, the X-ray diffractogram of this phase (Figure 13) shows at low angle a series of peaks corresponding to four orders of reflections of a lamellar structure with a repeat distance of 52 Å. In the wide-angle region, it shows only two peaks at 4.17 Å and 4.11 Å. These results are coherent with a reverse lamellar phase, with the alkyl chains in the outer layers, in contact with oil, and the glycerol head in the inner layer of the lamellae, and disordered alkyl chains. The distance of 4.17 Å is attributed to a 2D hexagonal order of the polar head of the glycerol. The gelation is due to this reverse lamellar structure. Indeed, in water, **MG** also forms a lamellar phase, but it does not gel the solvent. It is a direct lamellar phase: **MG** forms bilayers with the alkyl chains inside the bilayer, and the polar head in contact with water.

Below *T*_K_, the diffractogram of the sub-alpha crystalline phase shows, at low angle, the same series of peaks characteristic of a lamellar structure, but with a repeat distance slightly reduced to 50 Å. The wide-angle region exhibits more peaks, which are consistent with a better crystalline order of the alkyl chains and are attributed to an orthorhombic packing.

**Figure 14:**
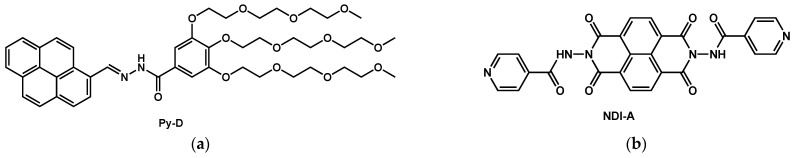
Chemical structures of (**a**) **Py-D** and (**b**) **NDI-A**.

Mixtures of the π-conjugated compounds **Py-D**, a pyrene-based donor, and **NDI-A**, a naphthalenediimide-based acceptor (Figure 14) form charge-transfer complexes in solution. In H_2_O:DMF (4:1 *v*:*v*), mixtures of **Py-D:NDI-A** in ratio of 1:1 and 2:1 form violet gels at room temperature [56]. These gels are named RT-gels.

**Figure 15:**
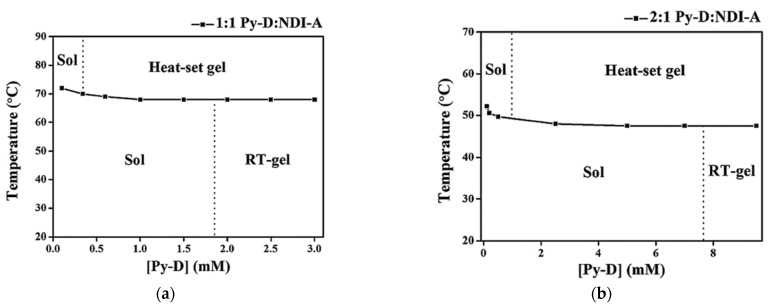
Phase diagram of **Py-D: NDI-A** in H_2_O:DMF: (**a**) 1:1 **Py-D: NDI-A**; (**b**) 2:1 **Py-D: NDI-A**. Reproduced from ref. [56] with permission from the Royal Society of Chemistry.

Solutions of **Py-D** alone in water show a lower critical solution temperature (LCST) and precipitate upon heating. The existence of this LCST impacts the phase behavior of the **Py-D**:**NDI-A**/H_2_O:DMF with an additional transition upon heating. The RT-gel is violet and translucent, and, when heated, it becomes cloudy. This gel is named heat-set gel. Surprisingly, when a **Py-D**:**NDI-A** solution at a concentration below the gel concentration is heated, it also yields a heat-set gel. The phase diagrams mapped for both studied **Py-D**:**NDI-A** ratios summarize these observations (Figure 15). TEM and SEM studies of the RT-gels and heat-set gels after drying evidenced different structures. The RT-gel is made of aggregated small globules (30–250 nm), whereas, in the heat-set gel, it is made of larger globules.

**Figure 16:**
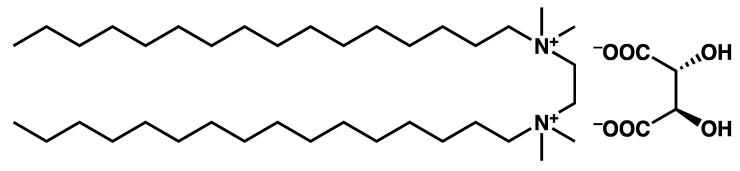
Chemical structure of 16-2-16 *L*-tartrate.

**Figure 17:**
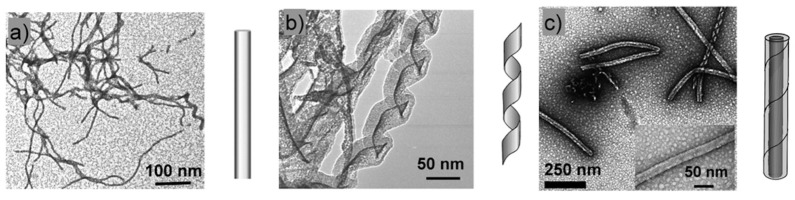
Evolution of the morphologies of the fibrous structures of **16-2-16 *L*-tartrate** (10 mM in H_2_O) over time. (**a**) After 2 h: fibers; (**b**) After 3 h: evolution towards helical ribbons; (**c**) After 36 h: formation of the tubules. Reprinted with permission from Ref. [57]. Copyright 2007 American Chemical Society.

In the previous examples, the gel-to-gel transition was triggered by the variation of temperature and was observed in a rather short time, for instance during a DSC measurement. Brizard et al. [57] have observed structural transitions in gels of **16-2-16 *L*-tartrate** (Figure 16) after ageing. The gels are formed by heating the sample above the gel-to-sol transition and cooling it between 21 °C and 24 °C. Their structure was studied by TEM at different times (Figure 17). Two hours after their formation, the gels are made of thin fibrils. After 3 h, the gels evolve to show helical ribbons, and after 36 h, the ribbons transit to closed nanotubes.

This transition from helical ribbons to tubules is also triggered by temperature. The gel-to-sol temperature transition of the studied sample was about 43 °C. When the sample is kept just below, between 38 °C and 40 °C, only ribbons form, and below 35 °C, the tubules form. When suspensions of tubules are heated back at 40 °C, they transit into ribbons after two months of equilibration. These results prove that the evolution is not a slow irreversible maturation, but corresponds to a reversible transition. This example is important because many molecular gels evolve after their formation and sometimes, it is necessary to wait for a maturation period to reach their final properties. This maturation process may correspond to a reversible transition. In the next section, we discuss some examples where this transition is not reversible.

### 2.2. Irreversible Transitions

**Figure 18:**
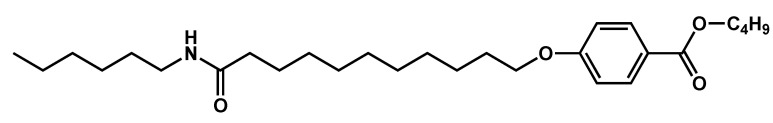
Molecular structure of **HUB-4**.

Gel-to-gel transition was observed in different systems upon ageing. **HUB-4** (Figure 18) forms gel in alkanes with concentration > 0.5 wt% in various alkanes [58].

**Figure 19:**
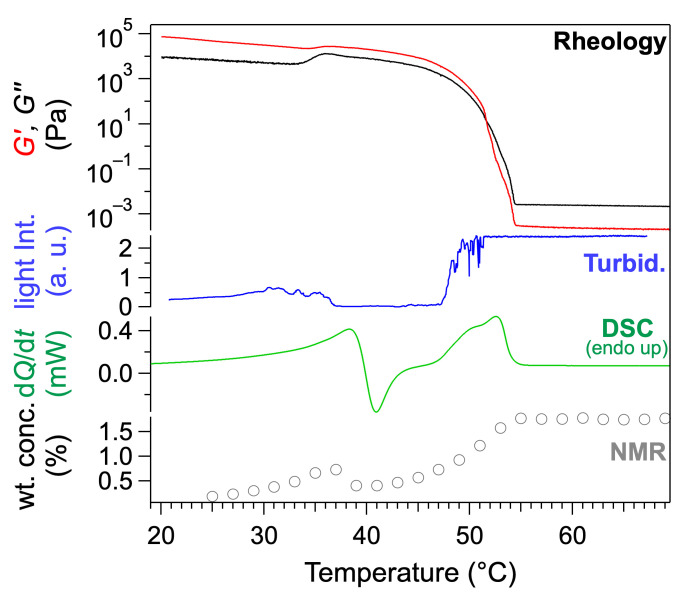
Rheology, turbidimetry, DSC, and NMR of **HUB-4**/*trans-*decalin upon heating (*c* = 2 wt%). All the techniques show two transitions: the sol-to-gel one at higher temperature (at 54 °C by rheology), and a gel-to-gel transition at lower temperature (at 37 °C by rheology). Reproduced from ref. [58] with permission from the Royal Society of Chemistry.

The transitions of the gel were studied over a decade of concentration by rheology, turbidimetry, DSC, and NMR. For concentration below 1 wt%, the gel-to-sol transition was observed by these techniques at the same temperature within experimental errors. At low concentrations, only this transition was detected. For concentration above 1 wt%, two transitions are observed as shown in Figure 19. The sol-to-gel transition is identified by rheology by the crossover of *G*′ and *G*″ at 53 °C. It corresponds to the end of the DSC endotherm at 54 °C, or to the beginning of the plateau of the NMR intensities at 54 °C. At a lower temperature, all the techniques show another transition. At 35 °C, both *G*′ and *G*″ increase, while *G*′ remains higher than *G*″, which shows that the sample transits from one gel to a different gel. This first transition was observed by different techniques in the same range of temperature. DSC shows an endotherm (max. at 38.3 °C) immediately followed by an exotherm (min. at 40.9 °C). These two events show the disassembly of a solid fraction, and the subsequent formation of another solid network. Finally, the NMR integrals increase up to 37 °C, where they decrease abruptly. The NMR integrals, when suitably renormalized, yield the soluble fraction of the gelator, which is the fraction not immobilized in the solid network [59]. The decrease at 37 °C thus indicates a decrease in the solubility of the gelators, and suggests that, at lower temperature, the mixture is supersaturated in gelator.

**Figure 20:**
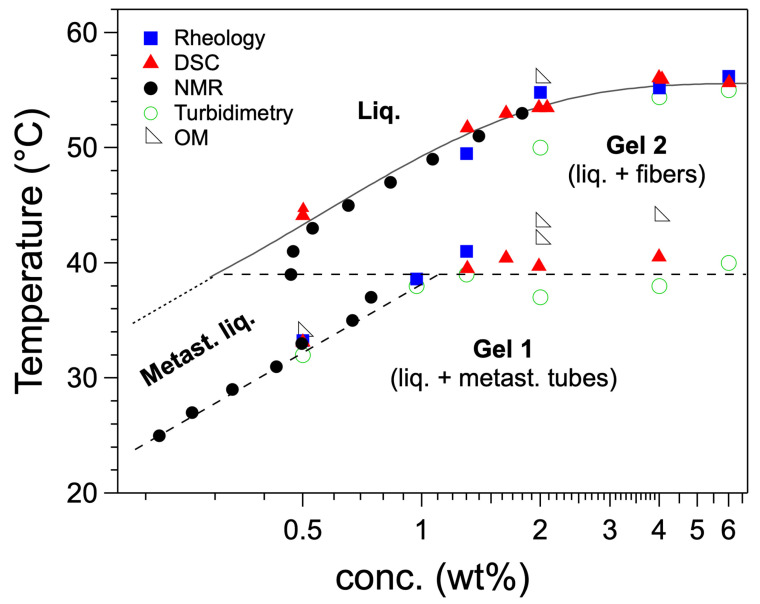
*c*-*T* phase diagram of **HUB-4**/*trans*-decalin. In this diagram, gel 1 is metastable, and the transition involving this gel (dashed lines) are irreversible. Reproduced from ref. [58] with permission from the Royal Society of Chemistry.

The *c*-*T* phase diagram of **HUB-4**/*trans-*decalin upon heating (Figure 20) was established over a decade of concentrations by the same techniques [58]. The structure and the relative stability of gel 1 and gel 2 were studied by SEM and X-ray scattering. Freeze fracture microscopy and SAXS show the freshly formed gel is made of nanotubes [60]. When the same gel is heated at 45 °C for 2 h, they disappear and are replaced by large fibers with widths comprised between 95 nm and 547 nm. The SAXS shows a disappearance of the signal of the nanotubes at low angle, and the WAXS region a series of Bragg peaks showing that the fibers are crystalline. When the gel is formed slowly (e.g., at—0.5 °C/min instead of 1 °C/min) the first transition is no longer observed: the observation of gel 1 depends on the thermal history of the sample. Gel 1 transforms into gel 2 simply by waiting a few days. This experiment shows that gel 1, made of nanotubes, is metastable, and that the gel 2 is the thermodynamically stable one. Therefore, in the phase diagram, the domain below the first transition represents the domain of existence of the metastable gel 1, but not a real domain of thermodynamic stability.

**Figure 21:**
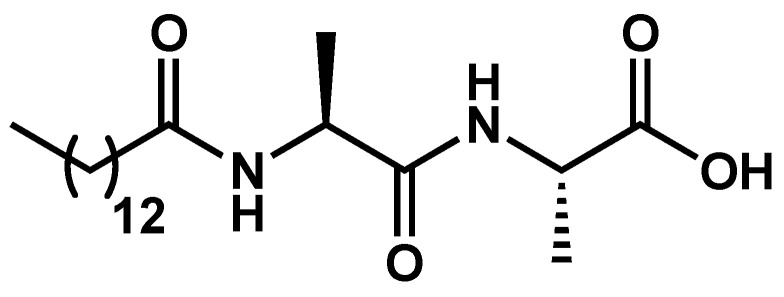
Chemical structure of **MAA**.

In the following example a gel-to-gel transition is observed upon ageing, but its triggering by temperature and its thermoreversibility have not been studied [61]. The dipeptide Ala-Ala *N*-acylated by a fatty acid (**MAA**, Figure 21) forms a hydrogel in the pH range of 7.0–8.5 at concentration of 3 mg/mL and higher.

**Figure 22:**
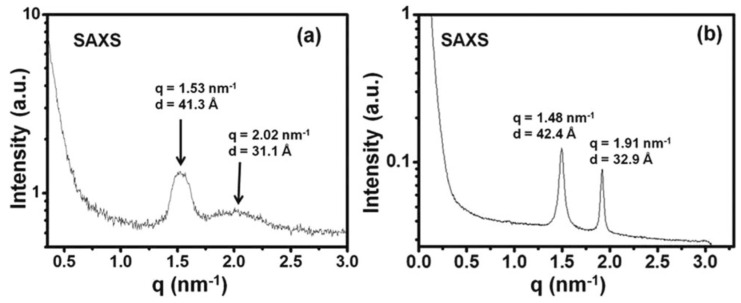
X-ray scattering of **MAA** hydrogel when (**a**) freshly formed and (**b**) aged for 2 days. Reproduced from ref. [61] with permission from the Royal Society of Chemistry.

The visual aspect of the gels changes with time. Gels at concentrations between 4 mg/mL and 5 mg/mL are transparent short after their formation, become turbid after 10 h and completely opaque after 2 days. SAXS intensities were measured for both fresh and aged gels. They showed peaks at the same angles, but better resolved in aged gels, which shows a superior crystalline order (Figure 22). The morphology of the gels was studied by SEM and TEM. Both techniques show a network of helical fibrils, both in the fresh and aged gel. The fibrils in both gels are very similar. The only difference is the length of the pitch of helices. In the aged gels, the pitch lengths are uniform. On the contrary, they are very polydisperse in the fresh gel.

In these two examples, **HUB-4**/*trans*-decalin and **MAA**/H_2_O, the irreversible gel-to-gel transitions is coincident with the apparition of Bragg Peaks, showing a better crystalline order. The gels forming faster correspond to less stable and less ordered forms but are kinetically favored. The gels formed after ageing are more stable and more crystalline forms but form slowly. These characteristics are common to the transitions studied in the next part.

## 3. Gel-to-Crystal Transitions

Organogels are made of interconnected particles, most often fibrils, in a solvent. In this section, we address the transformation of these fibrils into crystalline objects, such as crystals, spherulites, etc. These different forms are polymorphs of the gelator.

As an introducing remark, polymorphism has been observed already in early work on organogelators. As Lescanne et al. have shown [62], depending on the cooling rate, it is possible to orient the structure of the gel toward gels of fibrils or precipitates. The same authors have already surmised that the relevant parameter was the supersaturation rate. Wang et al. [63] also shown the gels of *N*-lauroyl-*L*-glutamic acid di *n*-butylamide can be composed of fibrils or spherulites, depending on the supersaturation rate. In this example, the cooling rate itself has no influence on the morphology, which confirmed Lescanne’s supposition. Huang et al. [64,65] studied a gel of steroid linked to a naphthyl group in alkanes. They show that, depending on the temperature at which the gel is formed and on the concentration, either a spherulitic or fibrous network is obtained. These examples show that the conditions of formation can orient the assembly of the gelator toward one of its forms, for instance fibrils, spherulites, or crystals.

**Figure 23:**
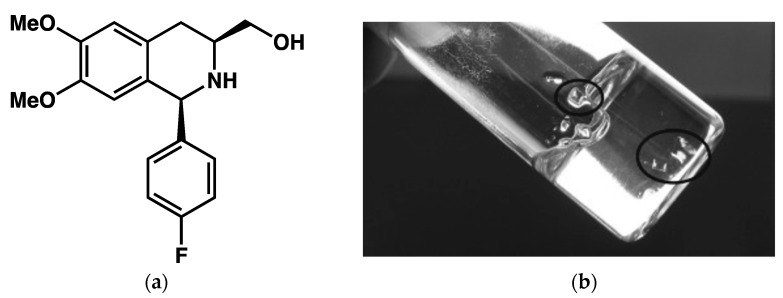
(**a**) Chemical structure of **B**; (**b**) Apparition of monocrystals in the gel of **B** in o-xylene. Reproduced with permission from ref. [66]. Copyright © 2023 WILEY-VCH Verlag GmbH & Co. KGaA, Weinheim.

Here, we focus on molecular gels for which one has observed the direct transformation of the gel to crystalline objects, such as spherulites or monocrystals. Most often, the transition occurs simply upon standing. A typical example is provided by the work of Xu et al. [66]. The amino alcohol **B** (Figure 23a) forms stable gels in various solvents. In *o*-xylene, gels are formed at 6 mg/mL, but are metastable, monocrystals appear in the gel matrix (Figure 23b), and eventually, the gel collapse to yield a flowing solution and crystals within 2 days.

The structure of the formed crystals was resolved by XRD, and showed that the unit cell contains two molecules of gelators and two molecules and *o*-xylene.

**Figure 24:**
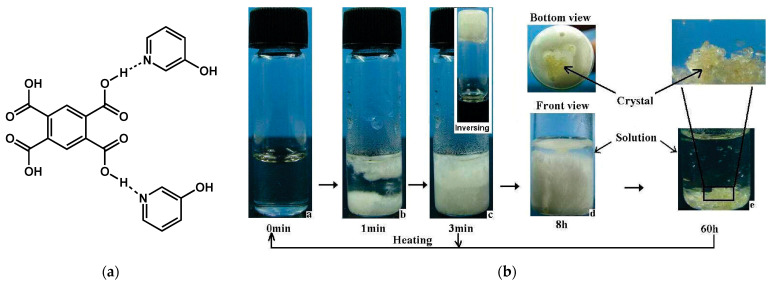
(**a**) Chemical structure of **G1**, prepared from 1,2,4,5-benzene tetra- carboxylic acid and 3-hydroxy pyridine at a molar ratio of 1:2.; (**b**) Evolution of the **G1** hydrogel (5 wt%) during the gel-crystal transition. Reprinted with permission from Ref. [67]. Copyright 2008 American Chemical Society.

Other examples of organogels lead to crystals suitable for crystallographic determination. For instance, **G1** [67] the *meta*-hydroxypyridinium salt of 1,2,4,5-benzene tetracarboxylic acid at a molar ratio of 1:2 (Figure 24a). **G1** gels water at concentration of 18 mg/mL with a melting temperature close to 35 °C. After 8 h, small crystals start to grow in the gel, and, after 60 h, the gel transforms into a suspension of crystals, which eventually sediment (Figure 24b).

**Figure 25:**
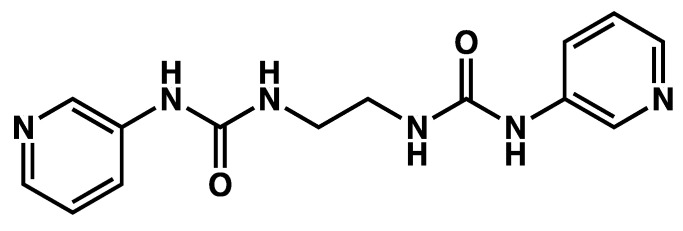
Chemical structure of dipyridyl urea **L^1^**.

Mixtures of the dipyridyl urea **L^1^** (Figure 25) and AgNO_3_ form gels in aqueous THF/H_2_O mixtures [68]. Many of these gels are not stable, and, upon standing, they transform into a clear solution and monocrystals, which are suitable for structure resolution by XRD. The same authors show similar results for other dipyridyl urea ligands.

**Figure 26:**
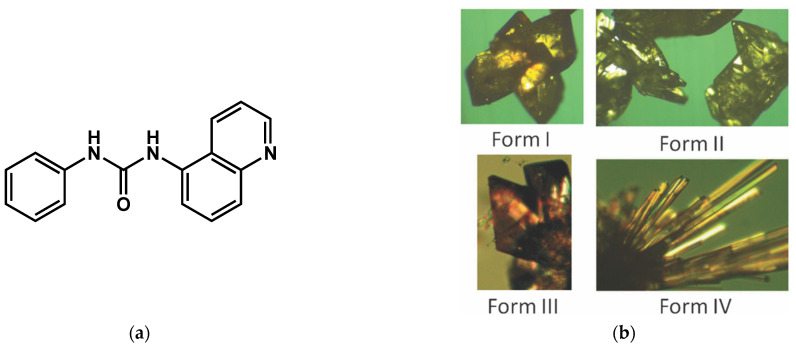
(**a**) chemical structure of **PQ5U**; (**b**) Images of each of the polymorphs of [Ag_2_(PQ5U)]NO_3_ obtained from gel-to-crystals transitions: from gels in CH_3_CN (form I), in EtOH (form II), in i-PrOH (form III) and in MeOH (form IV). Reproduced from ref. [69] with permission from the Royal Society of Chemistry.

Other gels of metal complexes, also called metallogels, were studied Braga et al. [69]. The gels are prepared form 1:2 mixtures of AgNO_3_ and the ligand **PQ5U** (Figure 26a) in CH_3_CN, MeOH, EtOH, or i-PrOH. The gels are transparent and after a few days transform into a clear solution and crystals of [Ag_2_(PQ5U)]NO_3_. Each solvent yielded a different polymorph (Figure 26b), and the crystallographic structure of these four forms were resolved.

**Figure 27:**
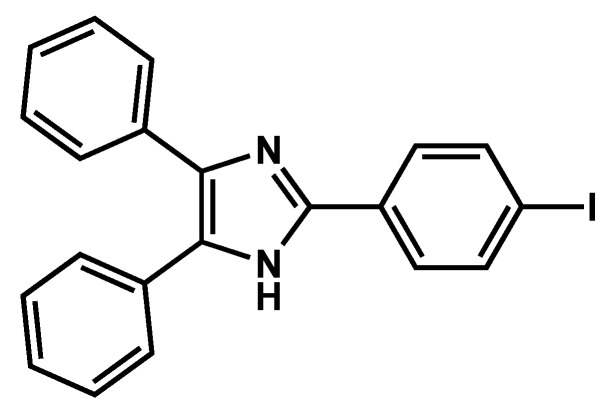
Molecular structure of the monoiodinated 2,4,5-triphenyl imidazole derivative **I-TPI**.

Such transitions leading to several crystalline forms that were observed by Andrews et al. [70] with **I-TPI** (Figure 27), an imidazole derivative gelling methanol with minimal concentration of 1.9 % *w*/*v*.

Upon ageing, the gels break down and large crystals form. The transformation occurs after irreproducible times: after a few days or after months. It can be accelerated by mechanical agitation: shaking, cutting, stirring, or an oscillatory shear. The structure of the crystal was resolved by XRD: they are a **I-TPI**:methanol 1:1 solvate [71]. They constitute a form named SI. The transformation is faster when water or DMSO are added to methanol in various proportions or when small compounds, such as 1,4-diiodobenzene, pyrene, 1-aminopyrene, are added in the sol. The formed crystals are also an **I-TPI**/methanol 1:1 solvate, but another polymorph named SII. With diiodobenzene, the mixture shows, simultaneously, SI, SII, and two additional polymorphs. These authors have estimated the packing energies of the different polymorphs and show that the most unstable forms form first. They have explained it by Ostwald’s rule, which states that the most unstable polymorphs are kinetically favored and form first; they convert later to the most stable forms. According to this rule, the molecular gel itself represents the first polymorph of the gelator.

**Figure 28:**
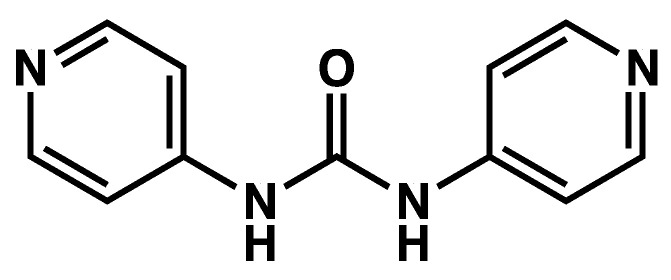
Chemical formula of **BPU**.

Kumar et al. also observed the formation of crystals from a gel of the *N*,*N*′-bis(4-pyridyl) urea **BPU** (Figure 28) in an ethyleneglycol/water mixture (1/9) [72]. After one month in an open test tube, crystals grow. The resolution of their structures by XRD revealed they are **BPU**·H_2_O·EG cocrystals.

**Figure 29:**
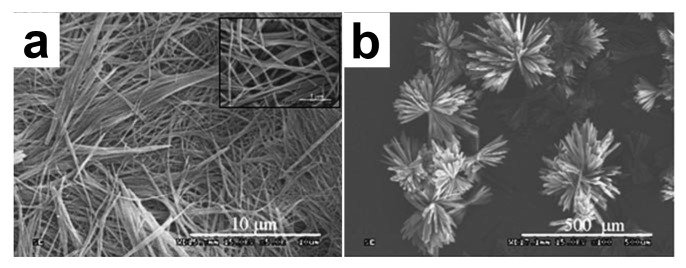
Observation by SEM of the self-assembled microstructures of dried samples, prepared at different ethanol/toluene ratios. Ethanol content: (**a**) 10 % and (**b**) 25 %. Reproduced from ref. [73] with permission. Copyright © 2023 WILEY-VCH Verlag GmbH & Co. KGaA, Weinheim.

In these examples, the gel-to-crystal transition produces monocrystals that were suitable for structure elucidation by XRD analysis. There are many other gels showing a transition to smaller crystallites or spherulitic structures. For instance, a transparent gel forms when a solution of the dipeptide phenylalanine-phenylalanine **FF** in 1,1,1,3,3,3-hexafluoro-2-propanol is diluted in toluene/ethanol mixtures [73]. With 10 % EtOH, the gel is an entangled network of fibers of 10 µm lengths (Figure 29a). With 25 % EtOH, a semi-transparent gel forms, and after 8 h, needle-like crystals grow with a flower-like morphology (Figure 29b). For 40 % EtOH, the gel no longer forms, but crystals appear after 10 min.

The intermolecular interactions were studied by FTIR. The gels made in pure toluene and in 10/90 ethanol/toluene, show a strong amide I’ band at 1683 cm^−1^ corresponding to a β-sheet structure. For higher ratios of EtOH, for which the crystals form, this band disappears, and the bands between 1650 and 1600 cm^−1^ are strongly modified, showing that the intermolecular interactions and the molecular arrays are different in the gel and the crystallites.

**Figure 30:**
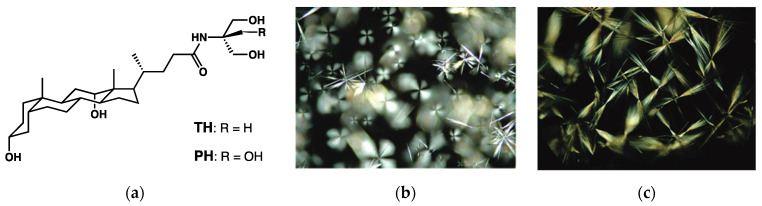
(**a**) Structure of the deoxycholic derivatives **TH** and **PH**; (**b**) **TH** gel (4.5 mg/mL) in 50% DMSO/H_2_O (1 h after preparation); (**c**) Crystals obtained from a **TH** gel (10 mg/mL) in 50% DMSO/H_2_O. Reprinted with permission from Ref. [74]. Copyright 2006 American Chemical Society.

Terech et al. [74] have shown the growth of similar crystalline structures in gels derivatives of deoxycholic acid **TH** and **PH** (Figure 30a) in DMSO/H_2_O or MeOH/H_2_O mixtures. These gels show the presence of spherulitic objects (Figure 30b), and after ageing, needle-like crystals grow from the spherulites, which act like nucleating seeds (Figure 30c).

A similar transition was observed for gels of a glycosylated amino acid derivative, Fmoc-Asp(Glc)-O*t*Bu [75]. Cubic crystallites of a few µm grow in the gels in CH_2_Cl_2_ or CHCl_3_. For gels in EtOH, fibrillar crystallites of several hundreds of µm form. Guterman et al. studied the gels of a pentapeptide (C-amidated FTLIE) [76] in water. When a drop of concentrated DMSO stock solution (100 mg.mL^−1^) is added into water, it forms a hydrogel which sediments in the solution. This gel was observed at 30 °C by optical microscopy. One hour after its formation, it showed a gel-to-crystal transition: the gel disappeared, and microcrystals grew.

**Figure 31:**
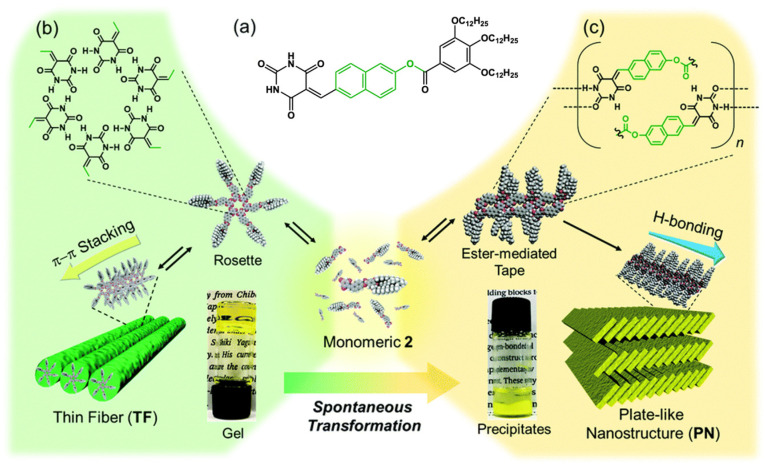
(**a**) Chemical structure of the studied compound; (**b**) photographs of the fresh gel in methylcyclohexane and scheme of the proposed molecular structure of the thin fibrils TF; (**c**) photographs of the precipitate after 10 h, and scheme of the proposed molecular structure for the precipitate PN. Reproduced from ref. [77] with permission from the Royal Society of Chemistry.

Barbituric acid coupled with a naphthalene derivative (Figure 31a) gels methylcyclohexane at concentration above 0.2 mM [77]. AFM studies showed that at 20 °C, the aggregates are bundle of thin fibers (TF) with a width of 6 ± 1 nm (Figure 31b). The gel is not stable; after 10 h, the sample becomes heterogenous, and after 12 h, it segregates into a solution and a precipitate (Figure 31c). The resulting suspension observed by AFM, shows no longer the thin fibers, but plate-like structures (PN) with a thickness of 3.64 ± 0.39 nm. FT-IR studies show that the thin fibers and the platelets have different H-bonds.

As shown by scanning tunnelling microscopy (STM), the platelets have a tilted structure. By comparison with the crystallographic structure of an analogue, the authors were able to propose a model of the molecular packing within these platelets, where the ester groups are H-bonded with the barbituric parts.

**Figure 32:**
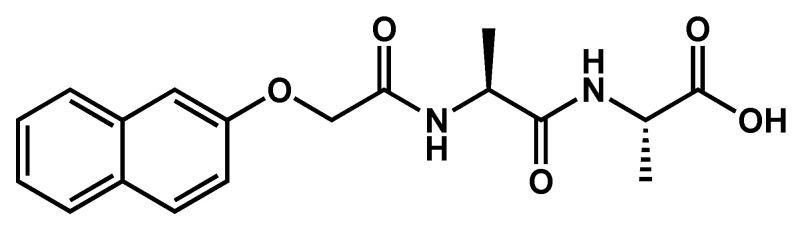
Molecular structure of **2NapAA**.

In most of examples of gel-to crystal transitions, the formation of the crystals results in the breakdown of the gel, which means that the initial network responsible for the viscoelastic properties disassemble and that the crystals grow at the expanses of this network. The evolution of both structures and their impact on the rheological properties have been carefully studied Giuri et al., thanks to a system where the kinetics can be tuned and slowed. The dipeptide alanine-alanine coupled to naphthalene **2NapAA** (Figure 32) gels aqueous solutions at pH ≈ 3.5 [78]. **2NapAA** is first solubilized at pH 10.5 (conc. 5 mg/mL). At this pH, the solution remains liquid. GdL (glucono-δ-lactone) is added to the solution. Its spontaneous hydrolysis into gluconic acid lowers the pH and results in the gelation of the solution. The resulting gels are metastable: crystals slowly form in the gel, and later, the gel turns into a liquid and some crystals. The rates of gelation crystallization increase with the amount of added GdL.

The gelation was followed by rheology experiments. For the lowest concentration in GdL, 4 mg/mL, the pH of the solution drops rapidly until it reaches 4.5 and diminishes slowly after this point. Then, close to the sol-to-gel transition *G*’ increases rapidly, and when the pH reaches 4.0, *G*′ plateaus at high values (100 kPa). For higher concentrations of GdL, the pH decreases faster. *G*′ and *G*″ increase, peak at pH 4.1, and decrease to become constant at values 3 orders of magnitude lower than with the gel formed at low GdL concentration (100 Pa or less). The variations of *G*′ for the higher GdL concentrations suggest a two steps process, consistent with the fast formation of the gel and its slower transformation into crystals.

The crystallization was studied by optical microscopy. For [GdL] = 4 mg/mL, large spherulitic domains (~0.7 mm) appear after 50 min, but with no crystals. For higher GdL concentrations, crystals with mm sizes appear with rates increasing with GdL concentrations.

**Figure 33:**
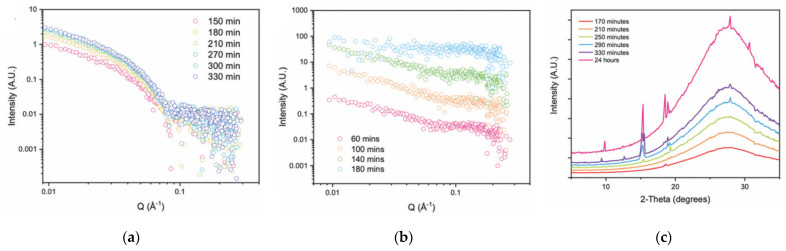
SAXS intensities for **2NapAA**/H_2_O (**a**) with concentration of GdL at 4 mg/mL. (**b**) 8 mg/mL (the intensities are shifted for clarity). (**c**) WAXS pattern for the solution with GdL at 8 mg/mL showing the evolution of the Bragg peaks. Reproduced from ref [78] with permission from the Royal Society of Chemistry.

The transformation was followed by SAXS and WAXS for two GdL concentrations, 4 and 8 mg/mL, respectively. For [GdL] = 4 mg/mL, the SAXS intensities (Figure 33a) could be fitted with a model of flexible cylinders with a radius of 2.9 nm at early stages of gelation and increasing to 4 nm after 210 min. No Bragg peaks were visible by WAXS. For [GdL] = 8 mg/mL, the SAXS intensities (Figure 33b) could also be fitted with the model of the cylinder with a radius varying from of 3.7 nm at the beginning to 3 nm after 170 min. Afterward, the SAXS intensities corresponding to the cylindrical fibrils disappear, Bragg peaks appear in the WAXS region, and their number increases with time (Figure 33c). This evolution corresponds to the apparition and growth of the crystals at the expanse of the fibrillar network of the gel, and explains the decrease in the elastic modulus.

**Figure 34:**
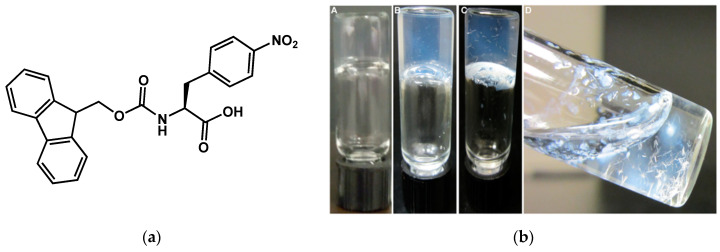
(**a**) Molecular structure of the **Fmoc-4-NO_2_-Phe**; (**b**) Aspect of the **Fmoc-4-NO_2_-Phe** hydrogel at different times after its formation. (**A**): 20 s, transparent; (**B**): 12 h; (**C**): 24 h appearance of a crystals in the gel; (**D**) 48 h, breakge of the gel, suspension of crystal in a liquid solution. Reprinted with permission from Ref. [79]. Copyright 2015 American Chemical Society.

**Fmoc-4-NO_2_-Phe** (Figure 34a) is another case where the transition was followed by structural studies [79]. This compound forms a transparent gel in DMSO/H_2_O mixtures. After a few hours, a precipitate appears in the gel. After 48 h, the hydrogel turns into a liquid suspension of needle-like macroscopic crystals (Figure 34b). Examination of these crystals by SEM showed they are hollow rods with square sections of 15–20 µm.

**Figure 35:**
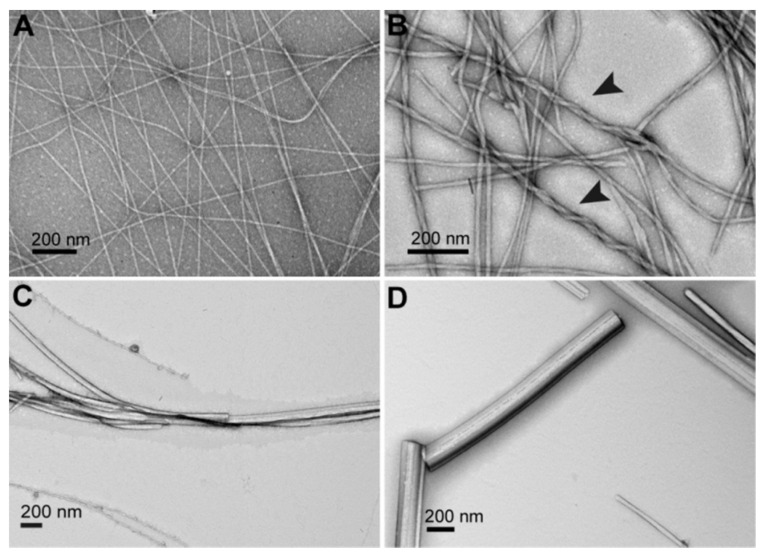
TEM observation of the fibril-crystal transition of the **Fmoc-4-NO_2_-Phe** hydrogel as a function of time: (**A**) 2 min, fibrils; (**B**) 10 min, clustering of fibrils; (**C**) 60 min; (**D**) 12–24 h. Reprinted with permission from Ref. [79]. Copyright 2015 American Chemical Society.

The progression of the transition could be followed by TEM all the way from the gel to the precipitate (Figure 35). The initial hydrogel shows a fibrillar structure with fibrils with diameters of 11.9 ± 2.0 nm and lengths of several micrometers (Figure 35A). After 10 min, the fibrils merge to form thicker fibrils (Figure 35B), and after 12–24 h, crystalline microtubes (Figure 35C,D). These micrographs show the evolution: from a network of numerous objects high aspect ratio, consistent with the gel state, to fewer objects with lower aspect ratio and less connected, which is consistent with the loss of the mechanical properties of the gel.

The crystallographic structure of the final crystalline microtubes was resolved by XRD. In this structure, the molecules interact with many interactions, π-π interactions (Fmoc-Fmoc and benzyl-benzyl) and H bonds between the carboxyl groups, leading to dimerization of the molecule. The authors have proposed for the fibrils in the initial gel structure, a structure of stacks of molecules with similar interactions, except the carboxylic groups are not paired, but in contact with the aqueous phase. With these models, the fibril-to-crystal transition corresponds to the reorganization of the fibrils by desolvation of the carboxyl groups and their H-bonding with each other.

The same group has taken advantage of this mechanism to stabilize the gels by chemical modification of the gelator [79]. For instance, varying the position of the nitro group of the phenyl group or esterifying the acid by triethyleneglycol prevents the transition to crystals. The same group has already shown that the pentafluorophenyl analogue forms hydrogels that form precipitates after a few weeks, and that precipitation is precluded with the corresponding triethyleneglycol ester [80,81]. This inhibition is consistent with the proposed mechanism, since it prevents the association of the carboxyl groups.

**Figure 36:**
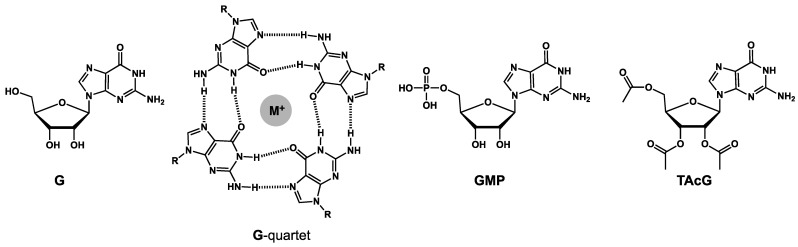
Structures of guanosine (**G**) and its assembly into G-quartets. Structure of guanosine derivatives, which stabilizes hydrogels when mixed with **G**: guanosine monophosphate **GMP** and tri-*O*-acetylguanosine **TAcG**.

The same approach, derivatization to slow and prevent the gel-to-crystals transition, has been implemented to hydrogels of guanosine **G** (Figure 36) [82,83]. In the presence of potassium or sodium salts, **G** self-assembles into G-quartets (Figure 36), that stack to form fibrillar aggregates, which are responsible for the gelation. The gels are not stable, and within a few hours, **G** crystalizes into the gel and eventually the mixtures flow.

However, Yu et al. have shown that, when **G** is mixed with 5′-guanosine monophosphate (**GMP**, Figure 36), the gels become more stable with higher temperatures of gel onset and reversible sol-gel transition [82]. Buerkle et al. have prepared aqueous gels with mixtures of tri-*O*-acetylatguanosine (**TAcG**, Figure 36) [83]. Ratios from 40/60 to 60/40 prevent the crystallization and the gel becomes stable.

**Figure 37:**
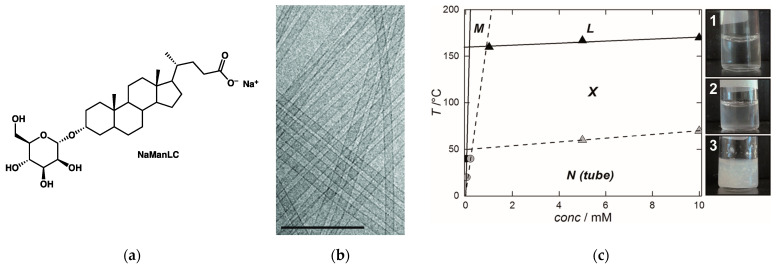
(**a**) Molecular structure of **NaManLC**; (**b**) TEM images of the nanotubes formed from **NaManLC**. Bar = 200 nm. Reproduced with permission from Ref. [84]. Copyright 2014 American Chemical Society; (**c**) Phase diagram of the **NaManLC**/NaOH aqueous system. L: isotropic solution of micelles, X: suspension of crystals, N: nematic solution of nanotubes, and M: solution of monomers (dissociated NaManLC). Grey and black circles: solubility values for tubules and crystals, respectively, measured by SLS. Grey triangles: transition of N to X measured by DSC; black triangles: dissolution of crystals (measured visually); (1) solution L, (2) suspension of crystals X, and (3) solution of nanotubes N. Reproduced from Ref. [85] with permission from the Royal Society of Chemistry.

In this section, we include the example of the lithocholate derivatives **NaManLC** (Figure 37a) [84,85] in aqueous NaOH, although it does not form a true gel, but a viscous transparent solution. As shown by cryo-TEM and SAXS, the compound self-assembles into nanotubes with external and inner diameters of 20.4 nm and 16.6 nm (Figure 37b) [85]. They form a nematic phase which shows birefringence. At around 60 °C, this suspension of nanotubes transforms into a turbid suspension of crystals. This transition is visible by DSC. When the suspension is heated, the crystals dissolve to yield a clear solution. The solubility of **NaManLC** in both forms was measured by light scattering. This allowed to map the phase diagram of **NaManLC**/aqueous NaOH (Figure 37c). The nanotubes transform irreversibly into crystals by sonication or by successive heating/cooling cycles. It shows that the nanotubes are metastable. They form at higher concentrations than crystals: they correspond to a supersaturated state, which is consistent with their metastability. The phase diagram, with its domain of metastable nanotubes and its domain of stable crystals, has a striking similarity with the phase diagram of **HUB-4**/*trans*-decalin studied above. In both cases, the nanotubes represent a metastable and supersaturated state.

## 4. Liquid–Liquid Phase Separation

**Figure 38:**
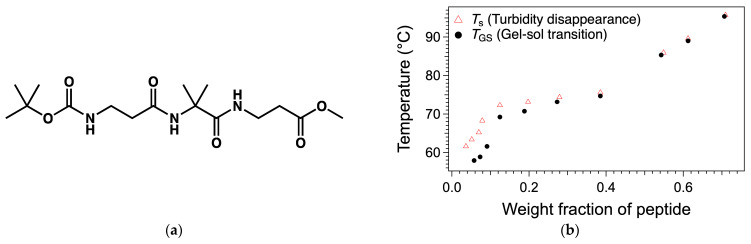
(**a**) Structure of the tripeptide **Boc-β-Ala-Aib-β-Ala-OMe**; (**b**) *c*-*T* phase diagram of **Boc-β-Ala-Aib-β-Ala-OMe**–dichlorobenzene. Plotted with data from ref. [86].

Malik et al. already described such a phase separation in organogel twenty years ago [86]. They studied gels of the tripeptide **Boc-β-Ala-Aib-β-Ala-OMe** (Figure 38a) in 1,2-dichlorobenzene (DCB). At a concentration of 1 wt%, no gel forms, but after 12 h, the authors observed a macroscopic phase separation, in two layers. For samples forming gels, at higher concentrations, they have studied the transitions by DSC and by visual observation. For weight fraction *W* < 0.27, upon heating, the sample starts to flow at *T*_GS_ and this sol is turbid. When heated a few degrees higher at *T_s_*, the sol becomes transparent. The sol between *T*_GS_ and *T*_s_ is turbid because it is a liquid-liquid biphasic system. Therefore, in the phase diagram (Figure 38b), the domain between *T*_GS_ and *T*_s_ defines a miscibility gap. *T*_s_ and *T*_GS_ superimpose for *W* ≥ 0.4. Therefore, depending on the concentration, the melting of the gel follows two different pathways: with a liquid–liquid phase separation at low concentration or with a monophasic sol at high concentration.

**Figure 39:**
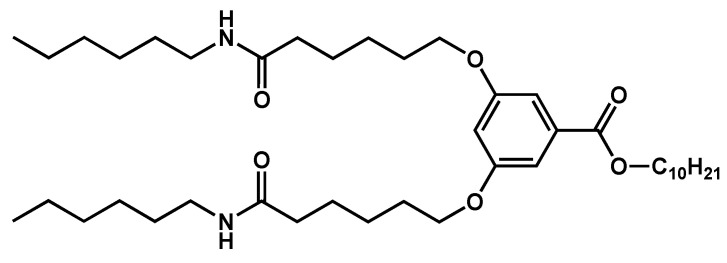
Chemical structure of **BHPB-10**.

**Figure 40:**
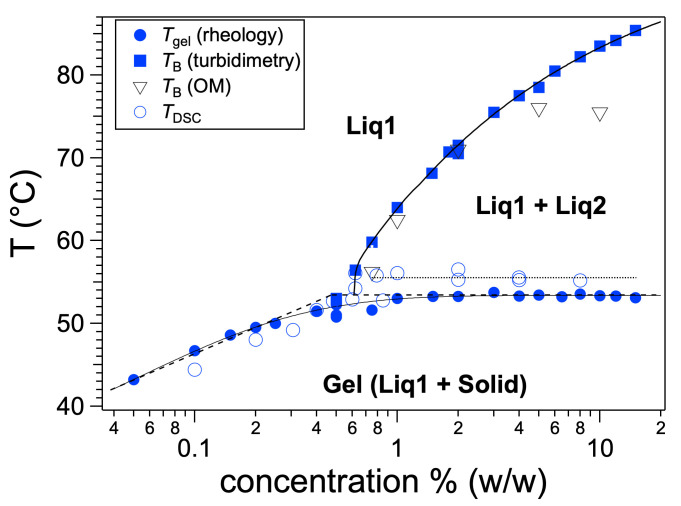
*c*-*T* phase diagram of **BHPB-10**/*trans*-decalin upon cooling (−0.25 °C/min). *T*_gel_: temperature of sol-to-gel measured by rheology. *T*_B_: temperature of liquid–liquid phase separation measured by turbidimetry or optical microscopy. Adapted with permission from Ref. [87]. Copyright 2016 American Chemical Society.

The diamide **BHPB-10** (Figure 39) forms gels in alkanes and aromatic solvents [87]. The phase diagram in *trans*-decalin upon cooling was established over a decade of concentration (Figure 40). The temperatures of the sol-to-gel transitions were measured by rheology and by DSC experiments. The temperatures measured by rheology are reported as *T*_gel_; the maximum temperature of the exotherm was reported as *T*_DSC_. Both temperatures show the same variation with concentration: at low concentration, *T*_gel_ and T_DSC_ increase with *T*. When the concentration cross *c*_L_ ≈ 0.6 wt%, over more than a decade, *T*_gel_ and *T*_DSC_ plateau, respectively at 53.1 ± 0.3 °C and 55.3 ± 0.4 °C.

**Figure 41:**
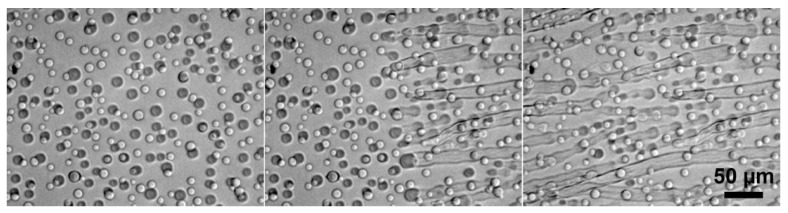
Optical micrographs of **BHPB-10**/*trans*-decalin mixtures (*c* = 2 wt%). Left: apparition of droplets at *T*_B_; middle and right: growth of the fibrils during the gel-to-sol transition at ~*T*_GS_. When the temperature is further cooled, only fibrils are visible. Adapted with permission from Ref. [87]. Copyright 2016 American Chemical Society.

In order to explain the existence of the plateau, the formation of the gel was followed by optical microscopy and turbidimetry at the same cooling rates applied for rheology and DSC experiments. At a high temperature, the sample is homogeneous, when the sample is cooled, droplets appear at a temperature called *T*_B_ (Figure 41 left).

X-ray scattering experiments on the biphasic mixtures confirmed that both the droplets and the continuous phase are liquid. Both liquids are solutions of **BHPB-10** in *trans*-decalin but with different concentrations. The concentration is higher in the droplets than in the continuous phase. When the temperature further decreases, the droplets start to disappear and fibers appear and grow (Figure 41 middle and right) and eventually the sample contains only fibers. The temperature *T*_B_ at which the liquid–liquid phase separation occurs can be measured also by turbidimetry. At high temperature, all the light intensity is transmitted through the sample. When the droplets appear, they scatter part of the light, which decreases the transmitted intensity. Below *T*_B_, the transmitted intensity is lower and constant, except around *T*_gel_, where it fluctuates. Turbidimetry thus detects the same two transitions as in optical microscopy: the liquid–liquid phase separation and the gel formation at lower temperature.

Above *c*_L_, the temperatures of gel-to-sol transition plateau. This constant temperature is simply explained by Gibbs’ phase rules. Above *c*_L_, the sol-to-gel transformation writes: liq 1 + liq 2→solid, so three different phases are in equilibrium; the pressure is fixed, which leads to a variance *v* = 0. This transformation is an monotectic transformation. Below the concentration *c*_L_, since only two phases are in equilibrium (liq→solid), *v* = 1, the temperature varies with the concentration. Inversely, the non-variance may reveal an equilibrium between three phases, hence a more complex transition. In the literature, some authors [88,89,90,91,92] described similar diagrams with two distinct regimes for the gel melting temperature: a continuous increase followed by a plateau. Such diagrams may also indicate a liquid–liquid phase separation.

**Figure 42:**
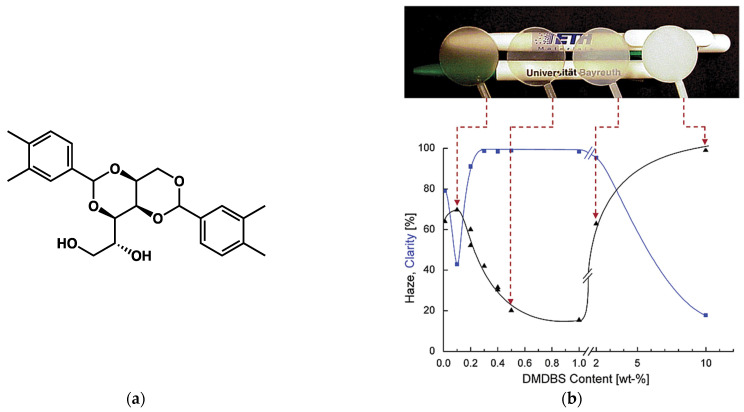
(**a**) Structure of **DMDBS**; (**b**) Variation of the clarity of ***i*-PP**/**DMDBS** mixtures as a function of the concentration of **DMDBS**. Reproduced with permission from Ref. [93]. Copyright 2003 American Chemical Society.

Liquid–liquid phase separation and monotectic transformations are also encountered in mixtures of gelators and polymers, where the gelator is used as a clarifying agent [93,94]. It has been shown that dibenzylidenesorbitol based gelators increase the clarity of semicrystalline polymers [95]. The polymer/gelator mixture is heated until it is fully melted. Upon cooling, the gelator forms a network of fibrils in the polymer melt and yields a physical gel. The fibrils are very efficient nucleating agents of the polymer and induce its crystallization upon further cooling, which increases the transparency of the solid polymer. Kristiansen et al. [93] have studied the clarity of mixtures of isotactic polypropylene (***i*-PP**) with the clarifying agent 1,3:2,4-bis(3,4-dimethyldibenzylidene)sorbitol (**DMDBS**, Figure 42a).

**Figure 43:**
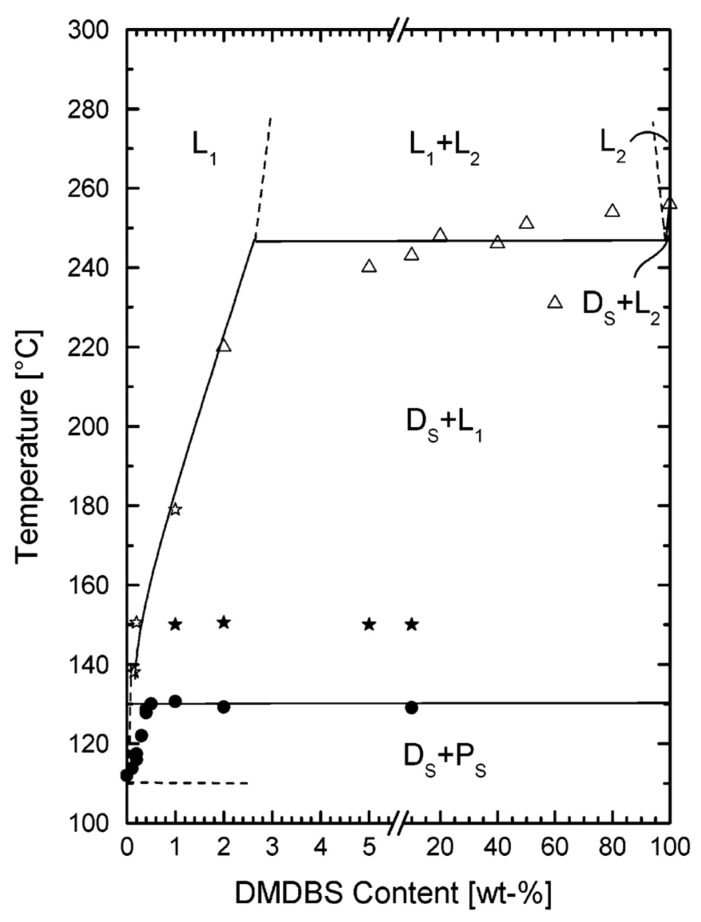
Temperature/composition phase diagram upon cooling of the ***i*-PP**/**DMBS** system. D_S_ refers to solid **DMDBS** and P_S_ to solid ***i*-PP**. L_1_ and L_2_ are liquids. The phase diagram was mapped with data obtained from DSC (•), optical microscopy (∆), and rheology (★/✩). The domain L_1_+L_2_ represents a liquid–liquid phase separation. Reproduced with permission from Ref. [93]. Copyright 2003 American Chemical Society.

This clarity of the sample is not linear with the concentration of the gelator (Figure 42b). It is optimal for a restricted range, between 0.2 and 1 wt% and diminishes for higher concentrations of **DMDBS**. This behavior was explained by the phase diagram of the binary system. In the optimal range of concentrations, when the mixture is fully melted, it forms a liquid (L_1_, Figure 43). Upon cooling, the gelator crystallizes and forms a solid network in the melted polymer (Ds + L_1,_ Figure 43). Therefore, Ds + L_1_ is a physical gel, where the liquid phase is the melted polymer. When it is further cooled, the polymer crystallizes to form the mixture Ds + Ps, where both the polymer and the gelator are solidified in separated solid phases.

For concentrations above 2 wt%, there is a liquid–liquid phase separation in the melt (L_1_ + L_2_, Figure 43). The transformation from this phase separated liquid-liquid domain L_1_ + L_2_ to the physical gel D_S_ + L_1_ is a monotectic transformation, and its temperature is constant, within the experimental errors, which is a consequence of Gibbs’ phase rule, as discussed above for **BHPB10**/trans-decalin. In the case of **DMDBS**/***i*-PP** mixtures, this monotectic transformation produces thick fibers, which reduce transparency and explains the upper limit of **DMDBS** concentration to improve clarity.

The lower limit of 0.2 wt% to yield transparent polymer corresponds to the eutectic point between the gelator and the polymer. Below this limit, the polymer crystallizes before the gelator, and is no longer nucleated by it.

Kristiansen et al. [94] have also studied another clarifying agent of ***i*-PP**, *N*,*N*′,*N*″-tris-isopentyl-1,3,5-benzenetricarboxamide and have obtained similar behavior and phase diagrams. These studies suggest that liquid–liquid phase separation is not rare in the field of organogels.

## 5. Eutectic Transitions

The ***i*-PP**/**DMBS** system studied above presents another non-variant transition: the crystallization of the liquid polymer from the gel to yield both solid components (D_S_ + L_1_ ➞ Ds + Ps). This transformation is a eutectic transition. Both components form separate solid phases. Eutectic transition has been reported in very early works by Terech for a steroid derivative in cyclohexane [96]. For molecular gels in regular organic solvents or water, if temperature was sufficiently lowered, the crystallization of the solvent should be systematically observed. However, the formed solid has no practical application, which explains the lack of interest to explore low temperatures.

**Figure 44:**
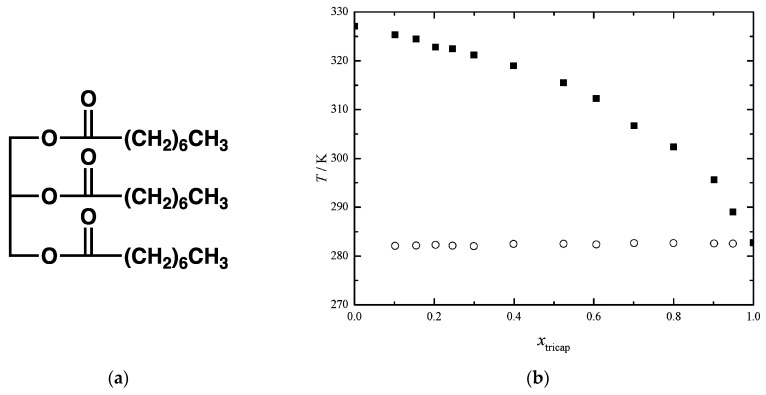
(**a**) Molecular structure of **tricaprylin;** (**b**) Phase diagram of the tetradecanoic acid/**tricaprylin** system. *X*_tricap_: molar fraction in **tricaprylin**; ■: melting temperature; ○: eutectic temperature. Reproduced with permission from Ref. [102]. Copyright 2010 American Chemical Society.

Eutectic transitions have been observed in oleogels. These are molecular gels in edible oils, which have developed as substitutes for solid fats in food products to lower the risk of cardiovascular diseases [97,98,99,100]. Eutectic transitions are rarely observed directly in these systems, where the solvent is a complex mixture of many triacylglycerols. However, simpler systems have shown eutectic transitions. For instance, edible oils can be gelled by fatty acids or fatty alcohol [101]. As Costa et al. have mapped the phase diagrams of model systems: mixture of various fatty acids in pure triacylglycerols [102]. The diagrams all show eutectic transformations. Figure 44b represents the example of tetradecanoic acid in **tricaprylin** (Figure 44a), where the eutectic transition can be observed at the constant temperature of 282 K. The same group has shown similar results for triolein/fatty acid mixtures [103].

Edible oils can be gelled by different waxes [104], e.g., Candelilla wax [105,106] or rice bran wax [107]. These waxes are mixtures of long linear alkanes and long alkyl fatty esters. Toro-Vazquez et al. have studied a gel of Candelilla wax in safflower oil, at 3 wt%. By DSC, they have observed the sol-to-gel transition at ~40 °C, and scanning at very low temperatures, they also observed an exotherm at ~–50 °C, attributed to the crystallization of triacylglycerols of the oil. Similar systems, mixtures of linear alkanes in C20, C24 and C28 in methyl stearate or methyl palmitate, studied by Benziane et al. [108] showed eutectic transitions. The determination of the composition and temperature of the eutectic has a fundamental interest. It allowed the authors to validate thermodynamical models (UNIFAC, Margules, etc.) predicting the solubility (or liquidus) of the gelator.

**Figure 45:**
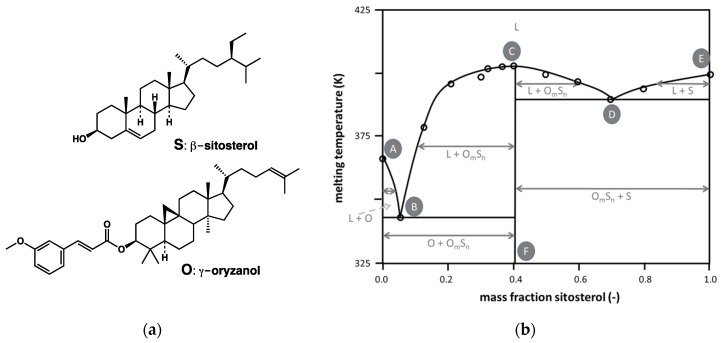
(**a**) Structures of β-sitosterol (**S**) and γ-oryzanol (**O**); (**b**) Simplified phase diagram of the binary mixture **O**/**S**. The line ABCDE is the liquidus. The line CF indicates the existence of a defined compound noted **O_m_S_n_**. B and D are eutectic points. Reproduced with permission from Ref. [112]. Copyright 2015 American Oil Chemists Society.

Finally, mixtures of β-sitosterol (**S**) and γ-oryzanol (**O**) (Figure 45a) are able to gel edible oils at a low wt% [109,110]. AlHasawi and Roger et al. have studied the phase behavior of the ternary mixtures of **S**/**O**/canola oil [111], Sawalha et al., and **S**/**O**/sunflower oil [112]. The latter have first mapped the phase diagram of the binary mixture **S**/**O** without oil. Figure 45b shows a simplified version of this diagram. It shows for a given composition a maximal melting temperature (C) surrounded by two eutectic points (B and D). It shows the existence of a compound **O_m_S_n_** with this composition.

**Figure 46:**
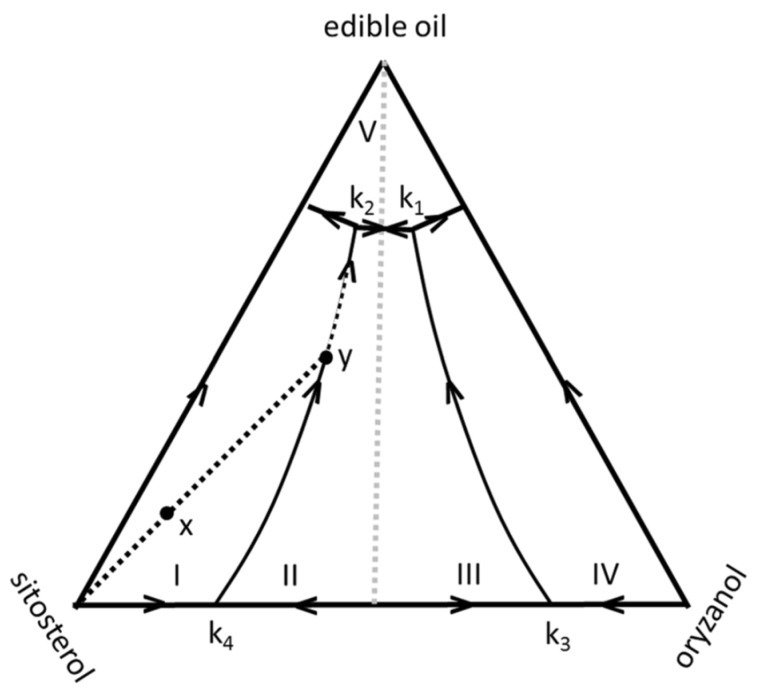
Projection of the melting surface of the ternary **S**/**O**/sunflower oil mixtures. Lines k_2_k_4_ and k_1_k_4_ connect the eutectic points of the binary diagrams. The line xyk_2_ is the solidification trajectory of a sample of composition x. The arrows on the border lines represent the evolution of composition of the liquid mixture upon cooling. Reproduced with permission from Ref. [112]. Copyright 2015 American Oil Chemists Society.

Figure 46 represents the projection of the melting surface of the ternary mixture. Its mapping assumes that each section, at constant oil composition (parallel to the **S**/**O** axis), is similar to the simplified binary phase diagram (Figure 45b). In this ternary diagram, the lines k_2_k_4_ and k_1_k_3_ join the eutectic points, equivalent to B and D in the binary phase diagram of **S**/**O**. The central regions II and IV are mixtures of solid **O_m_S_n_** with liquid oil, while the external regions I and IV are solid **O**/liquid oil and solid **S**/liquid oil, respectively.

In the ternary diagram, xyk_2_ indicates the crystallization pathway of a mixture of a given composition represented by the point x. In the first step, **S** crystallizes, and the liquid melt is enriched in **O** and oil. When the mixture is cooled further, it moves until the composition y, on the line of the binary eutectic points. At this point, both **S** and **O_m_S_n_** crystallize. This transformation is a eutectic transition. The system eventually reaches the ternary eutectic point k_2_, where all the components solidify.

## 6. Syneresis

**Figure 47:**
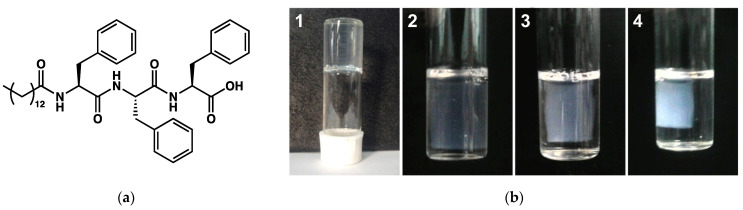
(**a**) Structures of *N*-tetradecanoyltriphenylalanine **MF**; (**b**) Images of the hydrogel of **MF** during syneresis obtained at different time intervals after formation: (1) 0 h, (2) 1 day, (3) 4 days, (4) 7 days. Reproduced from ref. [116] with permission from the Royal Society of Chemistry.

The syneresis is the expulsion of the solvent from a gel while that gel is contracting. This transformation is encountered for instance in mineral gels [113] or in food products, such as dairy products [114,115]. In the domain of molecular gels, this transformation has been reported in a few cases. It occurs most often upon resting. A typical example is given by the hydrogels of *N*-tetradecanoyltriphenylalanine (**MF**, Figure 47a) studied by Basak et al. [116]. These gels form at a pH comprised between 7 and 8.5, for concentration ≥ 1.2 mM. Immediately after their formation, the gels start to shrink and release water (Figure 47b). After 7 days, the gels have released about 80 % of their solvent.

The morphology of the gels was studied by SEM before and after syneresis. It showed the same network of fibers and no significant transformation. The authors have attributed the syneresis to the hydrophobicity of the gelators, which is composed of three phenylalanines, with aromatic rings. Indeed, the hydrophobic nature of the fibrillar network was demonstrated by the increase in fluorescence of ANS in the gel.

Other diphenylalanine derivatives, *N*-acylated by side chains with naphthyl or phenyl groups have been mentioned to form hydrogels evolving through syneresis [117]. Adams et al. have observed similar syneresis for hydrogels of the Fmoc protected dipeptides Fmoc-Ala-Ala, Fmoc Gly-Ala, and Fmoc-Gly-Gly [118].

**Figure 48:**
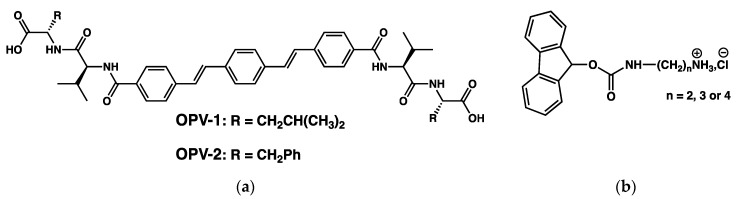
(**a**) Chemical structure of **OPV-1** and **OPV-2**. (**b**) Chemical structure of the **Fmoc-EDn** hydrochloride.

The same group has prevented the syneresis of a hydrogel by increasing the hydrophilicity of the self-assemblies of the gelators. They have first studied hydrogelators with an oligophenylenevinylene core appended to dipepetides, **OPV-1** and **OPV-2** (Figure 48a) [119].

These compounds were solubilized at pH 10 and the pH was lowered by adding glucono-δ-lactone (GdL), which resulted in a formation of a hydrogel in a couple hours. For instance, hydrogels of **OPV-1** at 5 mg/mL were formed from basic solution with GdL (5 mg/mL). The gelification starts when the pH reaches values lower than the pKa of the gelator, which is after about 1.5 h. The elastic modulus of the gel reaches its maximum after 4 h. Afterward, the gel starts to shrink, and after 18 h, expels about 60 % of its water. Gels of **OPV-2** also shrink, but to a less extent.

The authors formed gels of mixtures of **OPV-1** and a Fmoc monoprotected diamine (**Fmoc-DA**, Figure 48b) [120]. In an aqueous medium at pH 9.3, these mixtures are homogenous solutions, although at the same pH **Fmoc-DA** alone forms gels. It shows the existence of interactions between **OPV-1** and **Fmoc-ED**. When the pH is lowered, with the same amount of GdL as with the gel of **OPV-1** alone, it forms a gel and the elastic modulus reaches a stable value after comparable time (4 h), but no syneresis. **Fmoc-ED** and **OPV-1** interact and co-assemble, as proved by UV, fluorescence and FTIR experiments. Thus, this co-assembly increases the hydrophilicity of the network scaffold. It brings evidence that syneresis of molecular gels is driven by hydrophobicity of the self-assemblies, and not by a reorganization of the structure of the self-assembly at the molecular level.

**Figure 49:**
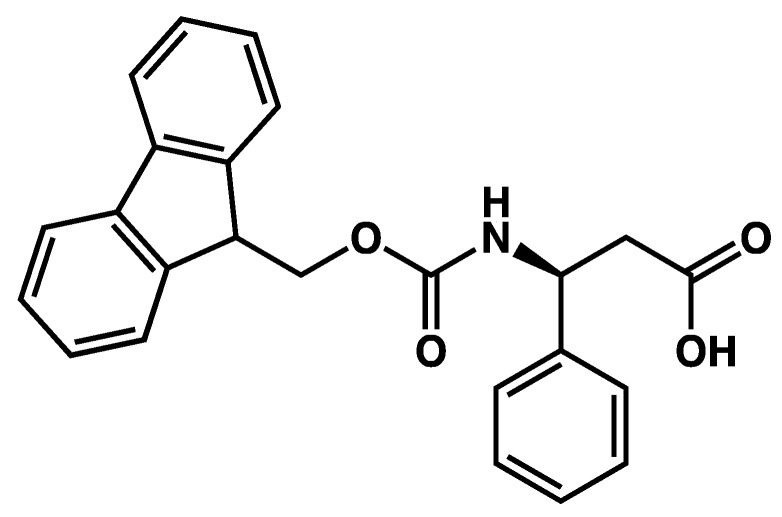
Chemical structure of **Fmoc-β-Phe**.

However, for some gels, structural studies have pointed out some differences in the organization of the gelators before and after syneresis. For example, the hydrogels of **Fmoc-β-Phe** (Figure 49), 30 min after their formation show syneresis [121]. In this case, the UV spectra show a blue shift of the absorption bands, attributed by the authors to a transformation of *J*- to *H*-aggregates. The FTIR spectra show no reorganization of the H-bonds after syneresis.

**Figure 50:**
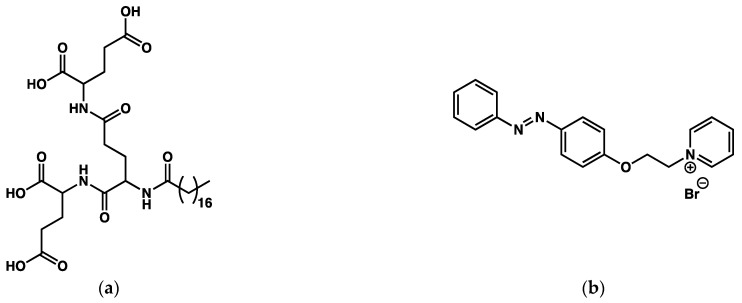
(**a**) Chemical structure of **OGAC**. (**b**) Chemical structure of **AZOC_2_Py**.

Xie et al. [122] have studied mixtures of an amphiphilic dendron terminated with three *L*-glutamic acid groups (**OGAC**, Figure 50a) and a positively charged azobenzene derivative (**AZOC_2_Py**, Figure 50b).

The mixture **OGAC**/**AZOC_2_Py** in water at a ratio of 5:1 (**OGAC** 0.13 wt%) forms a gel which slowly shrinks after its formation. After 12 h, the size of the gel reaches its equilibrium volume which represents a decrease of 60 % of the initial volume. UV spectra show that the expelled liquid contains **AZOC_2_Py**. The syneresis is accompanied by a modification of the CD spectra, which suggests a different molecular array arrangement of the gelator. AFM also shows an increase in the diameters of the fibers of the network, from 6.9 nm to 11 nm.

**Figure 51:**
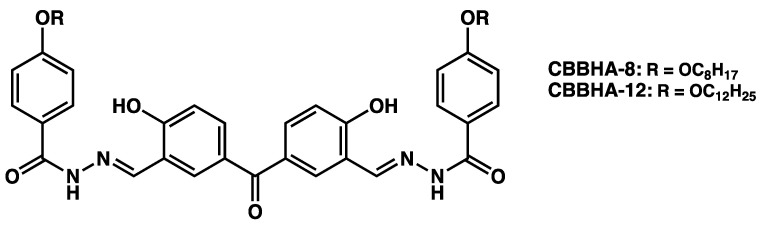
Chemical structure of **CBBHA-8** and **CBBHA-12**.

The same increase in the size of the fibers was observed by Ma et al. [123] with gels of **CBBHA-8** and **CBBHA-12** (Figure 51).

These compounds can gel 1,2 dichlorethane (DCE) at low concentrations, 2.29 µmol to 3.37 µmol/mL. The formed gels are stable for 12 h and then starts to shrink. In gels of **CBBHA-8**, after 3 to 5 days, the diameters of the fibers constituting the network have increased from 25–55 nm to 40–60 nm. WAXS experiments show a similar lamellar packing before and after syneresis, with a slight decrease in the lamellar spacing, 3.51 Å to 3.45 Å. At larger scale, the surface of the gels shows a pattern of circular areas, tens of micrometers wide, called honeycomb structure by the authors. This texturation is present in the fresh gel but disappears after syneresis.

Wu et al. have synthesized a complex gelator comprising two steroid units linked through a naphthalimide-amide spacer to a squaraine core [124]. It forms gels in toluene, when tris(2-ethanolamine) is present at molar fraction between 0.001 and 0.023. After its formation, the gel shrinks, and in two hours expels about 60% of the solvent. During the syneresis, the H-bonds of the system reorganize, as shown by FTIR. SAXS experiments evidence also a change from a lamellar structure to a hexagonal columnar system. The morphology of the systems was followed by confocal laser scanning microscopy. The fresh gel shows hollow spherical structures of 5–15 µm which, after 30 min, evolve into rod-like structures.

**Figure 52:**
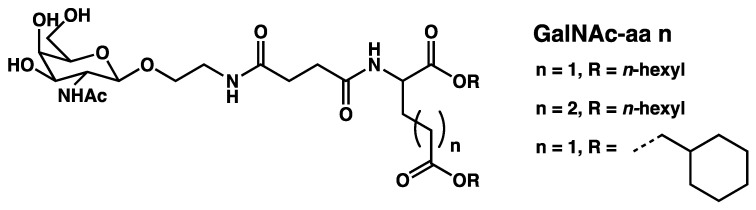
Chemical structure of the **GalNAc-aa** *n* gelators forming hydrogels with syneresis.

In the gels described above, syneresis occurs upon ageing, but there are examples where it is triggered by physical factors. The first example of syneresis in molecular gels, discovered by the group of Shinkai, was triggered by temperature [125]. It was observed with hydrogels of N-acetylgalactosamine appended aminoacids (**GalNAc-aa *n***, Figure 52).

**Figure 53:**
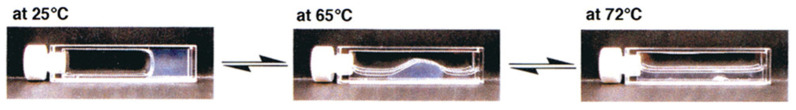
Aspect of a hydrogel of **GalNAc-aa 3** (4 mM, NaCl 250 mM) at different temperatures. At 65 °C, the liquid phase is partially expelled, and a liquid and the shrunken gel coexist. At 72 °C, the gel completely shrank to become a white precipitate, and more than 99 % of water was expelled. Reprinted with permission from Ref. [125]. Copyright 2002 American Chemical Society.

When a 4 mM hydrogel of **GalNAc-aa 3** is heated, it starts to shrink at 65 °C. At 72 °C, 99 % of the water is expelled and yields a white precipitate (Figure 53).

**Figure 54:**
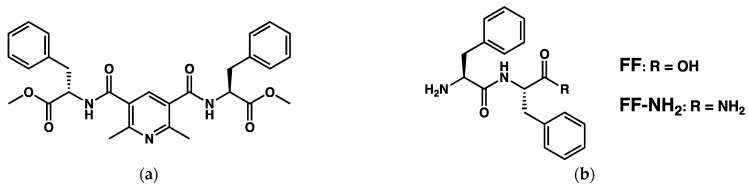
(**a**) Chemical structure of **P**. (**b**) Chemical structure of **FF** and **FF-NH_2_**.

When the shrunken gel is cooled back, it swells again and the hydrogel reforms. Gumtya et al. have shown that the gels of compound **P** (Figure 54a) in aromatic solvents, such as benzene, toluene, xylene, dichlorobenzene, are stable at room temperature, but exhibit syneresis when they are cooled at 15 °C [126].

Conte et al. have shown that the dipepetides **FF** and **FF-NH_2_** (Figure 54b) form hydrogels when solutions of this dipeptides in 1,1,1,3,3,3-hexafluoro-2-propanol (5 mg in 80 µL) in 1 mL in sodium phosphate buffer at pH 8 and sonicated [127]. The resulting hydrogels are stable at room temperature. However, mechanical contacts trigger their fast syneresis, resulting in the rapid expulsion of water and collapse into a semi-solid gel (the gel retains 40 % of the original volume). The authors observed no morphological change of the fibrillar network.

In conclusion, there are a few examples of molecular gels showing syneresis upon ageing or under a variation of temperature. The synereses in aqueous media are due to the hydrophobicity of the fibers, and structural and morphological changes of the self-assemblies are not necessarily observed. When syneresis is observed in organic solvent, it corresponds to deeper changes in the structure of the network.

## 7. Conclusions

This literature survey demonstrates that many molecular gels show additional transitions of other types besides the classical gel-sol transitions. Such transitions are detected and characterized by the same techniques implemented to study the structures of the gels. However, some of them, such as gel-to-gel or liquid–liquid phase separation, are less visible than the drastic change, from solid to liquid, observed during gel-to-sol transitions; they require more structural and DSC studies to characterize them. In the phase diagrams, the temperatures of gel-to-gel transitions, eutectic transitions or monotectic transformations are expected to be non-variant. Therefore, a constant melting temperature can indicate such transitions.

Part of the transitions described in this review are thermoreversible. As such they reflect the existence of additional stable phase domains in addition to one gel and one sol. For instance, a second gel domain or a liquid-liquid biphasic sol. These extra domains are stable, and as such, their formation can be easily controlled by concentration or temperature. While varying these parameters, crossing such a transition may result in an abrupt non-linear change of the properties, as shown by the example of clarifying agents/polymer mixtures. The knowledge and control of these transitions is integral for processing and applications.

Some of the observed transitions are irreversible, which indicates that the gel corresponds to transient metastable phases and evolves toward more stable phases. This is the case of the transformation of the gel into a suspension of crystals. Indeed, the reverse transformation is never observed directly: the gel can be reformed only via the sol after dissolving the crystals by heating. The network of the gel, most often fibrillar, may be considered like a metastable polymorph of the gelator. Andrews et al. [70] have made a very relevant connection of the gel-to-crystal transition with Ostwald’s rule: it shows that the gel represents the least stable polymorph, but is kinetically the most favored.

As seen in Section 2, some metastable gels transform irreversibly toward gels that represent more stable polymorphs and with a better molecular ordering, as shown by the apparition of Bragg peak, or transformation of thin to thicker fibrils. The mechanism is therefore the same than that of the gel-to-crystal trasnformation: the initially formed gel is an unstable polymorph and transits to a more stable and more crystalline one. The difference in the macroscopic state, gel or liquid suspension, just depends on the morphology of the final polymorph: if it forms particles with high aspect ratio and interconnected, the solution will remain a gel with solid-like behavior. On the contrary, if the formed solid particles have low aspect ratio, such as small crystals, and are not connected, no elastic network can form and the solution will flow.

This review has explored a few kinds of transitions reported in the literature. There may be other transitions, but they are not reported, probably because they occur in *c*-*T* domains, which are usually not explored. The most typical example is the eutectic transition, leading to the crystallization of the solvent, which should be systematically observed at low temperatures, but has no practical interest for applications. Such a transition is reported for polymers/gelator mixtures, because the crystallization of the polymer was central in the sought application. However, as shown in the case of oleogelators, the determination of the eutectic point has a fundamental interest since it provides a tool to validate thermodynamic models for the solubility of gelators. The same remark holds true for the identification of solvates, cocrystals or intercalates; it could be observed in a phase diagram, but at much higher concentrations than the few weight percent usually explored. These low concentrations are sufficient for the sought applications. However, exploring higher concentrations could unveil other types of transition and fundamental aspects on organogelators.

## Data Availability

Not applicable.
